# Thermally tunable naphthalene diimide solvates enable selective sensing, reversible photochromism, and anti-counterfeiting applications

**DOI:** 10.3389/fchem.2026.1760718

**Published:** 2026-03-04

**Authors:** Loveleen Kaur, Kawal Preet, Anasuya Mishra, Bigyan Ranjan Jali, Deepak B. Salunke, Subash Chandra Sahoo

**Affiliations:** 1 Department of Chemistry and Centre of Advanced Studies, Panjab University, Chandigarh, India; 2 Department of Chemistry, Government Autonomous College, Angul, Odisha, India; 3 Department of Chemistry, Veer Surendra Sai University of Technology, Burla, Odisha, India

**Keywords:** anti-counterfeiting, chemo-sensor, naphthalene diimides, photochromism, solvatochromism

## Abstract

Recent studies have highlighted the potential of naphthalene diimide (NDI)-based molecules for their stimuli-responsive and optical properties. In this work, we report two naphthalene diimide (NDI)-based solvates, designated as NDI-1 and NDI-2, were successfully synthesized from 4-aminopyridine and 1,4,5,8-naphthalene tetracarboxylic dianhydride (NDA) and comprehensively characterized using FT-IR, thermogravimetric analysis (TGA), UV-Vis, fluorescence spectroscopy, powder X-ray diffraction (PXRD), ^1^H NMR, single-crystal X-ray diffraction, and Hirshfeld surface analysis. Despite being solvates of each other, NDI-1 and NDI-2 exhibit distinct solvatochromic properties in DMF solution, as demonstrated by their unique UV-Vis absorption and fluorescence responses. Notably, the compounds display exceptional selectivity toward Hg^2+^ ions in DMF, outperforming a range of competing metal ions and producing characteristic optical changes. Furthermore, both solvates undergo reversible photochromic transformations upon exposure to UV light (365 nm), sunlight, and tungsten light, with the fastest switching under UV irradiation. These light-induced color changes gradually revert after removal of the stimulus, and similar reversible behavior is retained in polymer-embedded films (PVDF@NDI-1). Overall, this study provides a valuable strategy for designing multifunctional NDI-based materials, addressing the limited availability of conjugated systems that simultaneously exhibit multiple responsive properties and offering promising prospects for secure data storage and anti-counterfeiting technologies.

## 1 Introduction

Naphthalene diimides (NDIs) have emerged as a highly versatile class of π-conjugated organic materials, widely recognized for their exceptional thermal stability, strong electron-accepting character, and remarkable structural tunability (Al Kobaisi et al., 2016; Bhosale et al., 2008; Pan et al., 2011). Their rigid aromatic framework, coupled with the ease of incorporating diverse substituents at the imide or core positions, offers a powerful platform for engineering functional molecular architectures with tailored electronic and optical properties. Because of this inherent modularity, NDIs have become key building blocks in the development of advanced organic systems capable of responding to multiple external stimuli such as mechanical stress, acid, base, solvent, heat, and light. They have a wide range of applications in technologies related to sensors, anti-counterfeiting, optical storage, security encryption, and bio-imaging (Shi et al., 2023a; Shi et al., 2023b; Kalita et al., 2020; Li et al., 2024; Zawadzka et al., 2023).

Pyridine-based NDIs generate photoinduced electron transfer (PET) between electron donors and acceptors under photoexcitation, producing colored radicals. They remain widely utilized in the synthesis of multifunctional photochromic materials due to the ability of N atoms’ lone pairs to form stable chemical bonds with metal ions, leading to diverse crystal packing patterns that enhance the photoinduced electron transfer (PET) mechanism and functionality of naphthalene diimide-based crystalline hybrid photochromic materials (NCHPMs) (Han et al., 2013; Han et al., 2022; Li et al., 2025; Liu et al., 2014; Liu et al., 2015; Wei et al., 2018). Liu and Fu et al. have made great contributions to the synthesis and application of such NCHPMs (Liu J. J. et al., 2018). Coordination polymers (CPs) based on NDI linkers produce photochromic properties of MOFs (Hao et al., 2019; Liu et al., 2017; Liu J. J. et al., 2017; Liu J.-Jet al., 2022). In general, the photochromic properties of coordination polymers (CPs) originate from either structural transformations or photoinduced electron transfer within the photoactive organic ligands. These ligands can be chemically tailored and anchored into polymeric frameworks to endow the resulting materials with specific, desirable properties (Liu et al., 2018). Pyridyl-substituted NDI-based ligands are particularly versatile because they offer multiple coordination sites, including both terminal oxygen and nitrogen atoms. This diversity in coordination modes between the NDI ligands and metal cations not only enables the construction of a wide variety of networks but also allows fine-tuning of the photochromic behavior of the resulting CPs (Liu et al., 2021; Liu J.-J. et al., 2022; Liu J. et al., 2021). In addition, thermally tunable pyridine-based NDIs solvates are solid materials that co-crystallize with one or more solvent molecules that occupy channels, layers, or cavities formed by the NDI core stacking and pyridyl interactions.

In recent years, the exploration of thermally tunable NDI-derived molecules has gained significant momentum. Precise thermal control over molecular packing, polymorphism, or aggregation states can induce profound changes in the solid-state organization of these molecules, leading to distinct photophysical signatures. Such temperature-driven transformations enable reversible modulation of color, fluorescence, or absorption characteristics, positioning NDIs as excellent candidates for constructing intelligent materials with multi-responsive behavior (Liao et al., 2021; Li et al., 2023). These thermally mediated processes not only expand the fundamental understanding of organic solid-state dynamics but also offer valuable opportunities for designing adaptive materials with switchable optical properties (Liu et al., 2019; Liu J. et al., 2018; Liu et al., 2020). Among various sensing challenges, the selective detection of toxic heavy metal ions remains especially critical due to their persistence, bioaccumulation, and severe environmental impact. Designing molecular probes that offer high specificity, rapid response, and ease of operation is an ongoing research priority. NDIs, owing to their electron-deficient aromatic core and the possibility of fine-tuning their peripheral substituents, provide favorable coordination sites and efficient signal transduction mechanisms (Xu et al., 2025). Their strong charge-transfer capabilities and predictable structural organization make them particularly effective for selective sensing applications (Liu et al., 2025). Furthermore, recent literature reports indicate that NDI-based derivatives show unique photoresponsive properties and can be achieved in organic modifications. Although these examples are interesting, systems showing multi-dimensional properties, including polymorphic solvates, are hitherto unknown. In this context, thermally modulated NDI-based solvate systems exhibiting multi-responsive behavior present a promising strategy for realizing next-generation multifunctional materials. By integrating selective metal ion recognition, reversible photochromism, and temperature-induced optical switching within a single molecular framework, these materials can serve as efficient chemical sensors (Shi et al., 2024), smart optical switches, and robust anti-counterfeiting tools (Zhang T. et al., 2023; Shi Y. et al., 2023; Kalita et al., 2020; Yu et al., 2021).

The present work focuses on the rational design, synthesis, and comprehensive characterization of thermally tunable NDI-derived molecules with multi-responsive properties. Two para derivatives of naphthalene imide (p-NDIs) solvates (NDI-1 and NDI-2) were synthesized at two temperature conditions using reported procedures. Their structure–property relationships were established using various spectroscopic and analytical methods. Both solvates show unique photochromic behavior under light irradiations, solvatochromic properties, and selective sensing of Hg^2+^ ions in solution. Furthermore, the anti-counterfeiting properties of the NDI molecules were explored. Particular emphasis was placed on understanding how thermal modulation influences their structural arrangement, photophysical properties, and metal-ion sensing efficiency. The insights gained not only enrich the structure–property relationship of NDI systems but also establish a foundation for their practical implementation in environmental sensing, photonic devices, and security-oriented technologies.

## 2 Experimental

All the chemicals and solvents were commercially available and used without further purification. N,N-Dimethylformamide (DMF) was purchased from Fisher Scientific, while 4-aminopyridine and 1,4,5,8-naphthalene tetracarboxylic dianhydride (NDA) were purchased from Hyma Synthesis and GLR Innovations, respectively. ^1^H NMR spectra were recorded on a Bruker 400 Avance II spectrometer using TMS as an internal reference at room temperature. ATR/FT-IR spectra of all compounds were recorded using the Thermo Fisher Scientific Nicolet iS50 ATR/FT-IR spectrophotometer in the range 4,000–400 cm^−1^. Elemental analysis (CHN) was conducted on a Thermo Scientific Flash 2000 Organic Elemental Analyzer instrument. Thermal analysis of the NDI molecules was performed using the Mettler Toledo single differential thermal analysis (SDTA) sensor of the thermogravimetric analysis (TGA) instrument, in the temperature range of 25 °C–800 °C at a rate of 10 °C min^−1^ under a nitrogen atmosphere. UV-Vis spectra were obtained using a JASCO V-530 UV-Vis spectrophotometer. Powder X-ray diffraction (PXRD) was measured on an X’Pert Pro PANalytical instrument (*λ* = 1.5406 Å, Kappa, Anode–Copper). The measured parameter included a scan speed of 1 (°) min^−1^, a step size of 0.02 (°), and a scan range of 2θ from 5° to 50°. The steady-state absorption and emission spectra were recorded using the UV-Vis spectrophotometer (Shimadzu, UV-2600) and FluoroMax-4 spectrofluorimeter (Horiba Jobin Yvon), respectively. Fluorescence signals were collected at the magic angle using the MCP-PMT4S4 (Hamamatsu, Japan) detector.

### 2.1 Single-crystal X-ray crystallography (SCXRD) data collection

A single crystal of the synthesized compound NDI-1 was mounted using Hampton cryoloops. All geometric and intensity data for the crystals were collected using a SuperNova (Mo) X-ray diffractometer equipped with a micro-focus sealed X-ray tube (Cu-Ka) X-ray source, and a HyPix-3000 detector with increasing ω (width of 0.3 per frame) at a scan speed of either 5 s or 10 s per frame. CrysAlisPro software was used for data acquisition and data extraction. Using Olex2 (Dolomanov et al., 2009), the structure was solved with the SIR2004 (Burla et al., 2007) structure solution program using direct methods and refined with the ShelXL (Sheldrick, 2015) refinement package using least squares minimization. All non-hydrogen atoms were refined with anisotropic thermal parameters.

### 2.2 Hirshfeld analysis

The Hirshfeld surface studies were performed in CrystalExplorer 17.5 (Spackman et al., 2021; Turner et al., 2017). The Hirshfeld surface (HS) describes the boundary between the regions occupied by the central molecule and the surrounding neighboring molecules within a crystal lattice. This distinct surface enables analysis of intermolecular interactions in a crystalline environment, providing a unique fingerprint for studying various weak interactions. The presence of red colors on the Hirshfeld surface signifies the closest proximity between the surface and the adjacent atomic nuclei. Contact is established by adding the distance (d*i*) from the surface to the closest nucleus within the surface and the distance (d*e*) from the surface to the closest nucleus outside the surface. Significantly, d*e* is shorter than the combined van der Waals radii, emphasizing the importance of this interaction in the analysis of intermolecular forces.

### 2.3 Synthesis of 2,7-(dipyridine-4-yl) benzo[lmn] [3,8] phenanthroline-1,3,6,8(2H, 7H) tetraone (NDI)

**SCHEME 1:**
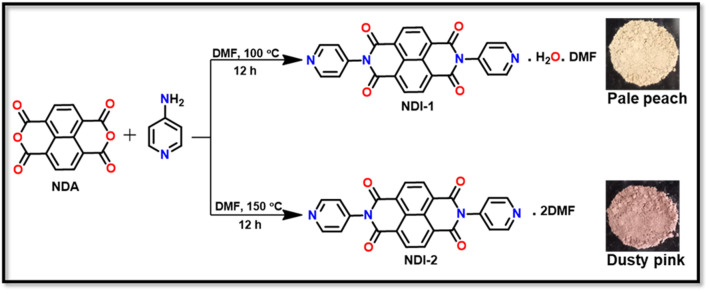
Synthesis of compound NDIs.

The synthesis of NDI-1 and NDI-2 was done by a modified literature procedure (Mizuguchi et al., 2005). 1,4,5,8-Naphthalene tetracarboxylic dianhydride (100 mg, 0.373 mmol) was taken in a round-bottom flask in 5 mL DMF. 4-Aminopyridine (100 mg, 1.11 mmol) was added to it as a solid to form a suspension. The reaction mixture was stirred for 12 h at 100 °C to obtain the desired product. Then, the reaction mixture was poured into an ice-cold water bath, filtered, and dried in an oven to get the pale peach-colored precipitates (NDI-1) (Scheme 1). Yield (78%). Anal Calcd. For C_27_H_20_N_5_O_6_ (510.48). C 63.46, H 3.91, N 13.72. Found: C 63.41, H 3.95, N 13.74. ^1^H NMR (400 MHz, CDCl_3_) δ (ppm) (Supplementary Figure S6): 9.17 (d), 9.15 (s), 8.37 (d), 3.35 (s). UV-Vis (DMF) λmax (nm) (Supplementary Figure S3): (a) 360, 381. (b) 537. ATR/FT-IR (νmax/cm^−1^) (Supplementary Figure S1): 1713 ν(asy, imide C=O), 1,681 ν(sy, amide C=O), 1,594 ν(DMF C=O). The product was crystallized in DMF. Alongside, the same reaction with similar quantities was carried out at 150 °C in DMF for 12 h. The precipitate obtained from this reaction was dusty pink and crystallized in DMF (NDI-2) (Scheme 1). Yield (76%). Anal Calcd. C_24_H_12_N_4_O_4_.2C_3_H_7_NO (566.57): C 63.54, H 4.58, N 14.82. Found: C 63.51, H 4.60, N 14.85. ^1^H NMR (400 MHz, CDCl_3_) δ (ppm) (Supplementary Figure S7): 9.16 (d), 9.14 (s), 8.38 (d), 3.36 (s). UV-Vis (DMF) λmax (nm) (Supplementary Figure S3): (a) 360, 380. (b) 537. ATR/FT-IR (νmax/cm^−1^) (Supplementary Figure S1): 1717 ν(asy, imide C=O), 1,683 ν(sy, amide C=O), 1,591 ν(DMF C=O).

## 3 Results and discussion

### 3.1 Synthesis and characterization

Two para-substituted naphthalene diimide-based compounds, named NDI-1 and NDI-2, were synthesized under different temperature conditions (100 °C and 150 °C) using NDA and para-aminopyridine using the procedure reported by Mizuguchi et al. (2005). The synthesized compounds can be easily distinguished based on their distinct colors: NDI-1—pale peach and NDI-2—dusty pink. Initially, it was not clear whether the origin of the different colors for NDI-1 and NDI-2 was due to impurity or different compounds. Surprisingly, after crystallization of both compounds in DMF solution, we found distinct crystal types. For NDI-1, we found a mixture of rod- and block-shaped crystals; for NDI-2 we found only rod-shaped crystals (Supplementary Figure S13). Each crystal type was examined for unit cell analysis using SCXRD before doing any bulk analysis. NDI-1 and NI-2 have a similar cell parameters, as reported by You et al. (2014), with CCDC numbers 1016732 and 1016731, respectively. However, we observed much-improved data for NDI-1 and, hence, updated the record in CCDC with a new number, 2512662, which was utilized in this present study for comparison.

The synthesized compounds were characterized using different spectroscopic and analytical techniques, including FT-IR, ^1^H NMR, UV-Vis, TGA, SCXRD, and a fluorospectrophotometer.

In ^1^H NMR, data for the pure crystals were taken in CDCl_3_. The appearance of peaks at 9.17 ppm and 8.37 ppm for NDI-1 and at 8.38 ppm and 9.16 ppm for NDI-2 confirms the formation of the diimides and corresponds to the Ar-H of the 4-aminopyridine ring (Supplementary Figures S6, 7).

FT-IR spectra for NDI-1 and NDI-2 were recorded and compared. The O-H frequencies of the compounds NDI-1 and NDI-2 were observed at 3,189 cm^−1^ and 3,218 cm^−1^, respectively, due to strong H-bonding interactions of the water molecule with the oxygen atom of the DMF and the nitrogen atom of the pyridine moiety. The imide C=O frequencies for both compounds appear at 1,713–1,715 cm^−1^ and 168–1,684 cm^−1^ for asymmetrical and symmetrical stretching, respectively. In addition, the stretching frequency for the amide C=O (of DMF) appears at 1,590–1,594 cm^−1^ (Supplementary Figure S1).

Thermogravimetric (TGA) measurements were performed on a TA Instrument SDT Q600 using alumina crucibles at a heating rate of 20 °C/min from 25 °C to 800 °C under a nitrogen atmosphere. NDI-1 and NDI-2 showed thermal stability with a decomposition temperature at 464 °C and 468 °C, respectively. The loss for NDI-1 was ∼16%, attributed to the loss of DMF and one water molecule, followed by complete decomposition of the molecule. The loss for NDI-2 was 13.5%, for the initial loss of one DMF, followed by the gradual loss of the remaining DMF (Supplementary Figure S2).

The UV-visible spectra of NDI-1 and NDI-2 were examined in N,N-dimethylformamide (DMF) solution, as shown in Supplementary Figure S3. Two major bands appear at ∼360 nm, 385 nm, and one shoulder at ∼341 nm, corresponding to the π → π*/n →π * transitions of the naphthalene moiety of both molecules in DMF solution (1 × 10^−5^ M) (Supplementary Figure S3a). Likewise, the band at approximately ∼380 nm also appears in the solid-state UV (Supplementary Figure S3b); however, a new band appears at 537 nm for both compounds in the solid-state UV, indicating that the compound behaves differently in the solid and solution phases.

A powder X-ray diffraction (PXRD) study of the finely powdered crystals and simulated powder X-ray patterns was compared with those obtained from SCXRD data. Peak-to-peak matching in major 2θ regions and the highly crystalline nature of the compounds revealed that both compounds can be synthesized in bulk (Supplementary Figures S4, 5).

### 3.2 Single-crystal X-ray crystallography (SCXRD) data collection

**FIGURE 1:**
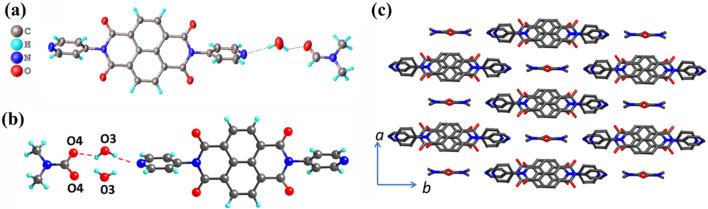
**(a)** Oak Ridge thermal-ellipsoid plot (ORTEP) diagram of the compound NDI-1 at 30% probability. **(b)** H-bonding interactions of the compound NDI-1 with solvents. **(c)** Lattice arrangement of NDI-1 along the c-axis.

Block-shaped, brown crystals of NDI-1 were grown by the slow evaporation of a DMF solution and successfully characterized by SCXRD. The compound is crystallized in the monoclinic space group of C2/c point group. The asymmetric unit of NDI-1 contains one-half of the molecule with one water molecule and DMF as a solvent of crystallization. The molecule, water, and the DMF molecule remain in the special position of the C2 axis and appear as half in the asymmetric unit. Both solvents were stabilized by strong H-bonding of 2.52 Å and 2.98 Å, respectively. The two side pyridyl groups are aligned slightly slanted to the central naphthalene diimide core. The pyridyl N-atoms form a basis for the H-bonding interactions toward the lattice solvents (Figure 1). Overall, the molecules form a lamellar motif, with 2D π-stacking along the *ab*-plane. The detailed description of the crystal structure is given in Supplementary Tables S2–4.

### 3.3 Hirshfeld surface analysis

The percentage of intermolecular interactions was further analyzed using the Hirshfeld surface (HS) and 2D fingerprint plots. Various types of interactions, such as C···C, C···H, H···H, N···H, O···H, O···C, C···N, O···N, and O···O in NDI-1 (Supplementary Figures S14–16) and C···C, C···H, H···H, N···H, O···H, O···C, C···N, O···N, O···O, and N···N for NDI-2 (Supplementary Figures S17–19) were identified from the 2D fingerprint plots generated via HS. These interactions can be used to determine the packing in the compounds by calculating (ρ), the ratio of C···H to C···C interactions. The ρ values for NDI-1 and NDI-2 were found to be 2.88 and 7.6, indicating a sandwich pattern for NDI-1 and a herringbone pattern for NDI-2.

## 4 Properties and applications

### 4.1 Solvatochromism

Naphthalene diimides (NDIs) have a strong electron-accepting character, high structural tunability, a rigid planar core, and the ability to incorporate diverse substituents, making them particularly attractive for designing functional materials. Among the various stimuli-responsive behaviors exhibited by NDIs, solvate formation has recently gained attention as a powerful strategy to modulate molecular packing, intermolecular interactions, and photophysical features. Solvate-assisted structural organization can induce distinct optical responses when exposed to different chemical environments, offering unique opportunities for selective sensing and chromic applications. It is well known that the solvatochromic behavior in molecules occurs when the electronic ground and excited states of the molecule are stabilized to different extents by solvent–solute interactions (Jha et al., 2023; Reichardt, 1994). When the excited state is stabilized more strongly in polar solvents, a bathochromic (red) shift is observed; conversely, when the ground state is stabilized more strongly, a hypsochromic (blue) shift is observed, as in the present study, where both compounds show a blue shift as the solvent polarity decreases.

**FIGURE 2:**
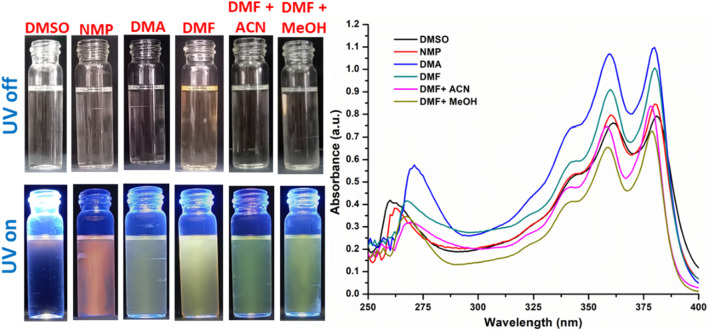
Solvatochromism in NDI-1 and corresponding UV absorption spectra.

**FIGURE 3:**
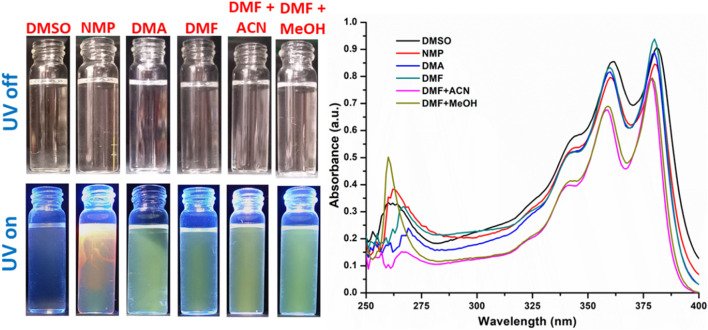
Solvatochromism in NDI-2 and corresponding UV absorption spectra.

Both NDI molecules exhibit pronounced solvatochromic behavior depending on the solvent environment. NDI-1 appears purple in DMSO, while in N-methyl-2-pyrrolidone (NMP), it shows a deep red color. In less polar solvents, the color shifts from light brown to pale yellow. Similarly, for NDI-2, the color also appears purple in DMSO, whereas it appears as red-yellow in NMP. The solvatochromic effect is significant only in DMSO and NMP, whereas in other solvents, the compound displays a green color with negligible variation for NDI-2 (Figures 2, 3).

The absorption spectra of NDI-1 show two maxima in the range of 360–385 nm. The absorption bands for both compounds show a hypsochromic effect (blue shift) with the decrease in the solvent polarity from DMSO to a mixture of various solvents. With decreasing polarity, DMSO > NMP > DMA > DMF > DMF + ACN (1:1)>DMF + MeOH (1:1), the absorption peaks shift from 362 nm to 358 nm and 381 nm to 379 nm. Similarly, for NDI-2, the blue shift occurs from 361 nm to 358 nm and from 381 nm to 378 nm.

Similar observations by Nikolaou et al. and others (Doria et al., 2013; Mateus et al., 2023) suggest that in polar solvents, π-stacking between N-(4-pyridyl)-1,8-naphthalimide (NI-py) molecules is thermodynamically favorable due to a preferable co-planar conformation between the pyridine and naphthalene units. In this environment, the NDI-py π→π* absorption band shifts to longer wavelengths with increasing solvent polarity (positive solvatochromism). Their analysis showed that the largest effect on this behavior was due to the solvent’s polarizability (Catalan’s SP parameter). At the same time, the solvent’s acidity also contributed due to the ability of the pyridine nitrogen and carbonyl oxygen to interact with the medium.

The present observation further encouraged us to explore the selective analyte detection and solvent-dependent chromic transitions. We hope that the findings provide important insights into structure–property correlations and pave the way for the future design of NDI-derived responsive materials with multifunctional applications.

### 4.2 Metal ion sensing

**FIGURE 4:**
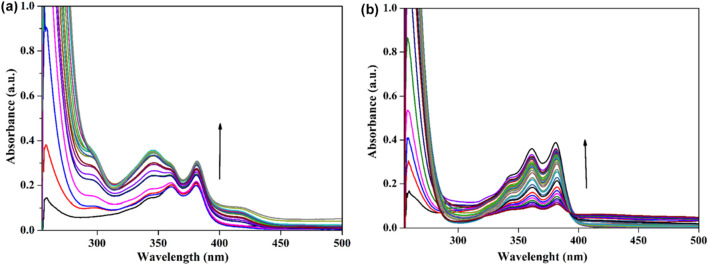
**(a)** UV-Vis spectra of NDI-1 (10^–5^ M, DMSO) in the absence and presence of Hg^2+^ ions (10^–5^ M, addition of Hg^2+^ ions 10 µL in each aliquot). **(b)** UV-Vis spectra of NDI-2 (10^–5^ M, DMSO) in the absence and presence of Hg^2+^ ions (10^–5^ M, addition of Hg^2+^ ions 10 µL in each aliquot).

NDIs bearing one supramolecular receptor unit at one of the two imide functionalities could be the best scaffold for binding complementary guests and could be easily monitored by titration experiments. Due to their high sensitivity at different concentrations, both binding constants in the lower range by ^1^H NMR and in the higher range (10^7^ M^−1^) are easily accessible by UV-Vis and fluorescence spectroscopy due to spectral shifts or changes in the fluorescence yield upon guest binding. With this aim, we have used the UV-Vis spectroscopic method to explore the interaction and binding nature of a synthesized compound probe and analytes (Praveen et al., 2020). The synthesized molecular probes (NDI-1 and NDI-2) were used to investigate the sensing behavior against various metal ions, such as alkali metal (Na^+^ and K^+^), alkaline earth metal (Mg^2+^ and Ca^2+^), transition metal ions (Mn^2+^, Co^2+^, Ni^2+^, Fe^2+^, Cu^2+^, Zn^2+^, Hg^2+^, and Cd^2+^) and p-block metal ions (Al^3+^) as their chloride and nitrate salts. The sensing behavior of NDI-1 and NDI-2 in DMSO was examined using UV-Vis and fluorescence spectrophotometry. The NDI-1 and NDI-2 probes selectively detected the Hg^2+^ ion over other metal salts (Figure 4). In DMSO, the NDI-1 and NDI-2 probes exhibited two absorption bands at 360 nm and 385 nm, respectively. The absorption bands are due to π→π* and n→π* transition processes. Upon interaction with various metal ions, the absorption bands did not change, except for the Hg^2+^ ions. The absorption bands at 360 nm and 385 nm were steadily enhanced upon the sequential addition of Hg^2+^ (10 µL in each aliquot). A similar observation was made in the case of NDI-2. This result demonstrates that the probe acts as an excellent colorimetric sensor for Hg^2+^ ions. To obtain more information about the nature of interaction and binding, the steady-state fluorescence titrations experiment has been explored and discussed below. The two distinct binding signatures in the solution spectra clearly indicate that separate structural entities, NDI-1 and NDI-2, exist within the solution state and that their interactions with the metal ions differ.

### 4.3 Fluorescence analysis in the presence of metal ions

**FIGURE 5:**
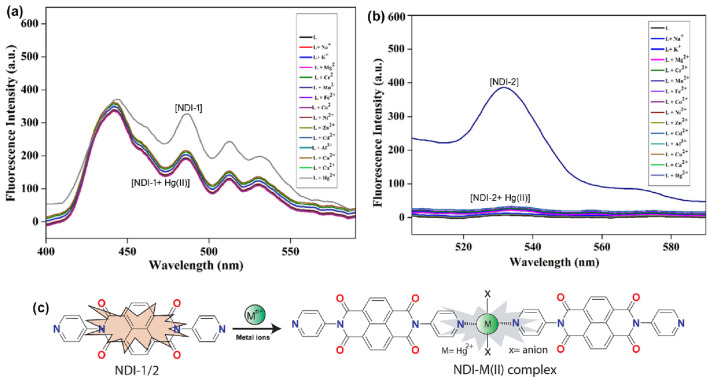
**(a)** Fluorescence spectra of NDI-1 (10^–5^ M, DMSO) in the absence and presence of Hg^2+^ ions (10^–5^ M, addition of Hg^2+^ ions, 10 µL in each aliquot). **(b)** Fluorescence spectra of NDI-2 (10^–5^ M, DMSO) in the absence and presence of Hg^2+^ ions (10^–5^ M, addition of Hg^2+^ ions, 10 µL in each aliquot). **(c)** Possible mechanism of sensing of NDIs toward Hg(II) ion in 1:2 stoichiometric ratio.

Fluorescence titration experiments were conducted to elicit the nature and interaction modes of the probe and analytes. Upon addition of Hg^2+^ ions to the DMSO solution, the fluorescence emission intensities steadily decreased (Figure 5a). These results revealed that our synthesized probe is highly selective for Hg^2+^ among numerous competing metal ions. The chloride and nitrate salts of metal ions, that is, alkali metals (Na^+^ and K^+^), alkaline earth metals (Mg^2+^ and Ca^2+^), transition metal ions (Mn^2+^, Co^2+^, Ni^2+^, Fe^2+^, Cu^2+^, Zn^2+^, Hg^2+^, and Cd^2+^), and p-block metal ions (Al^3+^) were used to evaluate the sensing ability of NDI-1 and NDI-2 in DMSO medium. Upon excitation at 380 nm, the NDI-2 probe exhibited a strong fluorescence emission band at 530 nm. NDI-1 shows four emission bands (Figure 5b). A fundamental photophysical characteristic of NDI-1 and NDI-2, photoinduced excited-state intramolecular proton transfer (ESIPT), is affected by DMSO, which may account for this strong emission peak. Theoretically, the ESIPT occurs through the formation of intramolecular hydrogen bonds between the proton donor and acceptor, which is preferred in nonpolar fluids. Because intramolecular and intermolecular hydrogen bonds compete with one another, the ESIPT is inhibited in polar solvents such as DMSO. Upon the addition of Hg^2+^ ions, fluorescence emission increased. These observations indicate the formation of a stable complex between the probes and Hg^2+^ ions (NDI-1 and NDI-2 + Hg^2+^). On the other hand, under identical conditions, no significant spectral or color changes were observed in the presence of other metal ions. This indicates the formation of a complex between the probe and Hg^2+^ ions (Figure 5c).

To further validate the selectivity toward Hg^2+^, Pb^2+^ was also evaluated as a representative competing ion, as both are soft/borderline-soft Lewis acids with comparable coordination preferences. It is surprising to note that the fluorescence emission intensities did not alter in the presence of Pb^2+^, as shown in Supplementary Figure S8. The fluorescence titrations demonstrated that both probes selectively detect Hg^2+^ ions over other tested metal salts. Though both Hg(II) and Pb(II) are relatively large heavy metal ions, the Hg^2+^ ion often acts as a soft acid, has higher complexation properties, and forms more stable complexes than Pb(II) primarily because its metal–ligand bonds possess a greater degree of covalent character, especially with soft ligands like sulfur and nitrogen (Paul et al., 2014). The existence of both soft (pyridyl N-atoms) binding sites is responsible for both probes’ strong selectivity toward Hg^2+^ over other metal ions.

### 4.4 Photochromism and anti-counterfeiting property

Recently, NDI-based solvates have emerged as promising candidates for the design of photoresponsive materials due to their ability to undergo reversible structural or electronic changes upon irradiation (Li et al., 2025). Such photochromic behavior typically manifests as a change in color or absorption characteristics under light exposure. It arises from solvated molecular arrangements that promote intramolecular charge transfer, generation of radical anions, or reversible conformational modifications. These characteristics make NDI solvates interesting platforms for advancing applications in molecular switches, optical memory devices, photo-actuators, and stimuli-responsive sensors (Zhang S. et al., 2023; Maniam et al., 2019).

**FIGURE 6:**
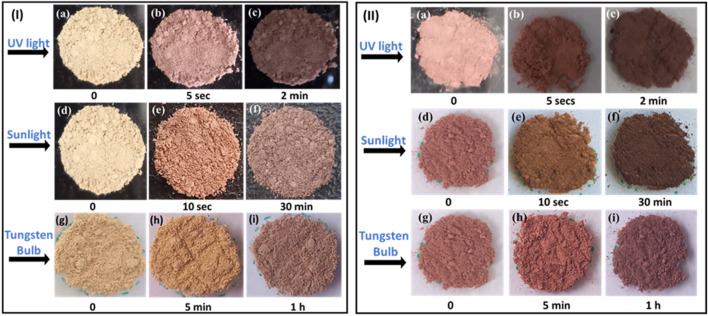
**(I)** Photochromism in NDI-1 under **(a–c)** UV light, **(d–f)** sunlight, and **(g–i)** tungsten bulb. **(II)** Photochromism in NDI-2 under **(a–c)** UV light, **(d–f)** sunlight, and **(g–i)** tungsten bulb.

Photochromic studies of NDI-1 and NDI-2 used powder that was finely ground using a mortar and pestle and irradiated under different light sources, such as UV light (365 nm), sunlight, and a tungsten bulb. Within 5 s of exposure to UV light, the pale peach color of NDI-1 began to change and became saturated after 2 min of illumination. In sunlight, a similar transition occurs at approximately 30 min, and the final-colored intensity was less dark than that achieved with UV-light exposure. Under a tungsten bulb, color changes take place after 50–60 min, and ∼1 h was needed to attain similar photochromic changes (Figure 6I). It is clear from the trend that under a UV-light source, photochromic changes are more profound and rapid, at least in the case of a normal bulb. The intensity of the UV-light source plays a crucial role. To determine the reversibility of the photochromic properties, the compounds were tested under switching of the light sources. Compounds were kept in the dark at room temperature. The irradiated products showed reversibility and returned to their original state, but the NDI-1 samples required more time (2 h each for the UV and sunlight-irradiated products and 3 h for the tungsten bulb) to return to baseline (Supplementary Figure S9). The above photochromic studies were also performed using pure crystals of NDI-1, and similar outcomes were observed, as shown in the figure in Supplementary Figure S10. The UV-Vis study reveals that upon irradiation, the effect on NDI-1 crystals is not enhanced absorption in the regions 200–400 nm, but rather the appearance of new bands with an absorption maximum at 635 nm (Supplementary Figure S11). These results suggest that the photoresponsive behavior originates from an electron transfer process within the framework. Thus, it is attributed to the photoinduced electron transfer transition rather than from a structural transformation (Al Kobaisi et al., 2016; Liu et al., 2015).

When a similar experiment was performed on the NDI-2 crystals, a similar trend was observed, although the end product of the NDI-2 transition was much darker.

For example, the color of NDI-2 also exhibits photochromism in every case. The color of the compound changes from dusty pink to brown (Figure 6II). However, the transition requires 5 s under UV light, approximately 10–20 s in sunlight, and 1 hour under a bulb to change its color. The compound also exhibits reversible photochromism in this case. It returned to its initial color within 1–3 h for all cases (Supplementary Figure S12). Additionally, the once-tested samples display color changes after being irradiated again, indicating that this photoinduced coloration–discoloration is also reversible.

Inspired by the promising sensing capabilities observed in both the solution and crystalline forms of NDI molecules, we fabricated a polymer-based composite film, PVDF@NDI-1, incorporating compound NDI-1 into a polyvinylidene difluoride (PVDF) matrix by the solution drop casting method (Supplementary Scheme S1). The solution casting method was chosen over others due to low cost and ease of fabrication. The crystal-to-polymer ratio was optimized to obtain a desired homogeneous film, and the film retains its flexibility as seen by mechanical force-induced bending. When stress is applied, the films bend easily without showing any fatigue (Supplementary Figure S20). The photochromic behavior of the composite film was tested under UV light. The film immediately changed its original color (pale peach) to light brown within 5 s and became dark after 2 min. After switching off the UV source, the film returned to its original color within 1 h, showing the reversible nature of the composite film (Supplementary Scheme S1).

**FIGURE 7:**
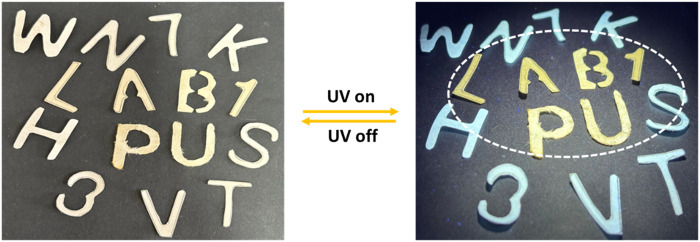
Anti-counterfeiting property of the NDI-1.

The photochromic properties of ND1-1, along with its quick responsive nature under UV, prompted us to explore its suitability for anti-counterfeiting applications (Figure 7) (Haider et al., 2022; Ma et al., 2024; Wan et al., 2023). Initially, a set of jumbled characters was cut out using pure Whatman filter paper. The selected characters (L, A, B, 1, P, and U) were immersed in a DMF solution of NDI-1 and air-dried. Before UV irradiation, all characters looked alike and were not distinguishable. However, after UV-light (365 nm) irradiation, characters L, A, B, 1, P, and U began to glow and emitted a pale yellow color under UV light, distinctively visible to the naked eye. This experiment has been repeated several times with the switch-on-off mode without any significant change in the color of the character strips.

## 5 Conclusion

In conclusion, we have synthesized and fully characterized two naphthalene diimide (NDI)-based solvates prepared from 4-aminopyridine and 1,4,5,8-naphthalene tetracarboxylic dianhydride (NDA), designated as NDI-1 and NDI-2. These materials were investigated using various analytical and spectroscopic techniques, including FT-IR, TGA, UV-Vis, fluorescence spectroscopy, PXRD, ^1^H NMR, single-crystal X-ray diffraction, and Hirshfeld surface analysis. Both solvates exhibit exceptional multi-responsive behavior, such as solvatochromism, selective metal ion sensing, photochromism, and anti-counterfeiting capabilities. Although NDI-1 and NDI-2 are solvates of each other, each displays distinct photophysical characteristics in solution, as supported by UV-Vis and fluorescence studies. Importantly, both derivatives show high selectivity toward Hg^2+^ ions in DMF, outperforming various competing metal ions and producing characteristic optical responses. The compounds also demonstrate reversible photochromic activity when exposed to different light sources, including UV light (365 nm), sunlight, and tungsten illumination, with the fastest response observed under UV light, moderate response under sunlight, and the slowest under tungsten light. The photoinduced color changes revert back once the light source is removed, although the recovery process is relatively slow. Similar reversible photochromism was further observed in polymer-composite films (PVDF@NDI-1). Overall, both materials hold strong potential for applications in secure data storage and anti-counterfeiting technologies. Given the scarcity of conjugated systems exhibiting multiple stimuli-responsive behaviors, this work offers a valuable approach for designing multifunctional materials with promising practical utility.

## Data Availability

The original contributions presented in the study are included in the article/Supplementary Material; further inquiries can be directed to the corresponding author.
