# Supplementary material for: Thermally tunable naphthalene diimide solvates enable selective sensing, reversible photochromism, and anti-counterfeiting applications
Source: Front Chem. 2026 Mar 4;14:1760718. doi: 10.3389/fchem.2026.1760718 (PMC12997075; doi:10.3389/fchem.2026.1760718)
Supplement: Supplementary file 1 [file DataSheet1.docx]

**Thermally Tunable Naphthalene Diimide Solvates Enabling Selective Sensing, Reversible Photochromism, and Anti-counterfeiting Application**

Loveleen Kaur^1^, Kawal Preet^1^, Anasuya Mishra^2^, Bigyan Ranjan Jali^3^, Deepak B. Salunke^1,4^, Subash Chandra Sahoo^1*^

^1^Department of Chemistry and Centre of Advanced Studies, Panjab University, Chandigarh, India

^2^Department of Chemistry, Government Autonomous College, Angul, Odisha, India

^3^Department of Chemistry, Veer Surendra Sai University of Technology, Burla, Sambalpur, Odisha, India

^4^Department of Medicinal Chemistry, National Institute of Pharmaceutical Education and Research, SAS Nagar, Punjab, India

**TABLE OF CONTENTS**

| **No.** | **FIGURES** | **Page No.** |
| --- | --- | --- |
| **Figure S1** | FT-IR Spectra of **NDI-1** and **NDI-2** | 2 |
| **Figure S2** | Thermogravimetric analysis of **(a)** **NDI-1** and **(b)** **NDI-2**. | 2 |
| **Figure S3** | **(a)** UV-visible data in Solution state of **NDI-1** and **NDI-2. (b)** UV-visible data in solid state of **NDI-1** and **NDI-2.** | 3 |
| **Figure S4** | Powder X-ray Diffraction patterns of Simulated **NDI-1** and synthesized **NDI-1** | 3 |
| **Figure S5** | Powder X-ray Diffraction patterns of **NDI-1** and **NDI-2** before and after irradiation. | 4 |
| **Figure S6** | **^1^**H NMR of **NDI-1** | 4 |
| **Figure S7** | **^1^**H NMR of **NDI-2** | 5 |
| **Figure S8** | Fluorescence studies of ligands with Pb^2+^ ion. | 5 |
| **Figure S9** | Reversible photochromism in **NDI-1** | 6 |
| **Figure S10** | Photochromism and its Reversibility in **NDI-1** crystals. | 6 |
| **Figure S11** | Solid state UV absorption data for **NDI-1** crystals before and after irradiation. | 7 |
| **Figure S12** | Reversible photochromism in **NDI-2**. | 7 |
| **Figure S13** | Optical images for **(a)** **NDI-1 (a)** and **(b)** **NDI-2** | 8 |
| **Figure S14** | **(a)** Hirshfeld surface analysis of **NDI-1**. **(b)** Hirshfeld 2D fingerprint plot of **NDI-1**. | 10 |
| **Figure S15** | **(a-f)** 2D fingerprint plots of **NDI-1** to obtain quantitative non-covalent interactions outlined from Hirshfeld surface analysis. The grey part in 2D finger plots indicates the total interactions calculated from *d_norm_*. | 10 |
| **Figure S16** | **(a-c)** 2D fingerprint plots of N–C**,** O–N**,** and O–O for **NDI-1** to obtain quantitative non-covalent interactions. The grey part in 2D finger plots indicates the total interactions calculated from *d_norm_* | 11 |
| **Figure S17** | **(a)** Hirshfeld surface analysis of **NDI-2**. **(b)** Hirshfeld 2D fingerprint plot of **NDI-2**. | 12 |
| **Figure S18** | **(a-f)** 2D fingerprint plots of **NDI-2** to obtain quantitative non-covalent interactions outlined from Hirshfeld surface analysis. The grey part in 2D finger plots indicates the total interactions calculated from *d_norm_*. | 12 |
| **Figure S19** | **(a-c)** 2D fingerprint plots of N–C**,** O–N**,** O–O, and N–N for **NDI-2** to obtain quantitative non-covalent interactions. The grey part in 2D finger plots indicates the total interactions calculated from *d_norm_* | 13 |
| **Figure S20** | The composite fil **PVDF@NDI-1** showing mechanical flexibility under mechanical stress. | 14 |
|  | **TABLES** |  |
| **Table S1.** | Major FT-IR Vibrations of **NDI-1** and **NDI-2** | 2 |
| **Table S2.** | Crystal Data and Structure refinement for the **NDI-1** | 8-9 |
| **Table S3.** | Selected bond lengths (Å) for the compound **NDI-1** | 9 |
| **Table S4.** | Selected bond angles (Å) for the compound **NDI-1** | 9-10 |
|  | **SCHEME** |  |
| **Scheme S1.** | Schematic Representation for preparation of **NDI-1** doped in PVDF film and its photochromic studies. | 14 |


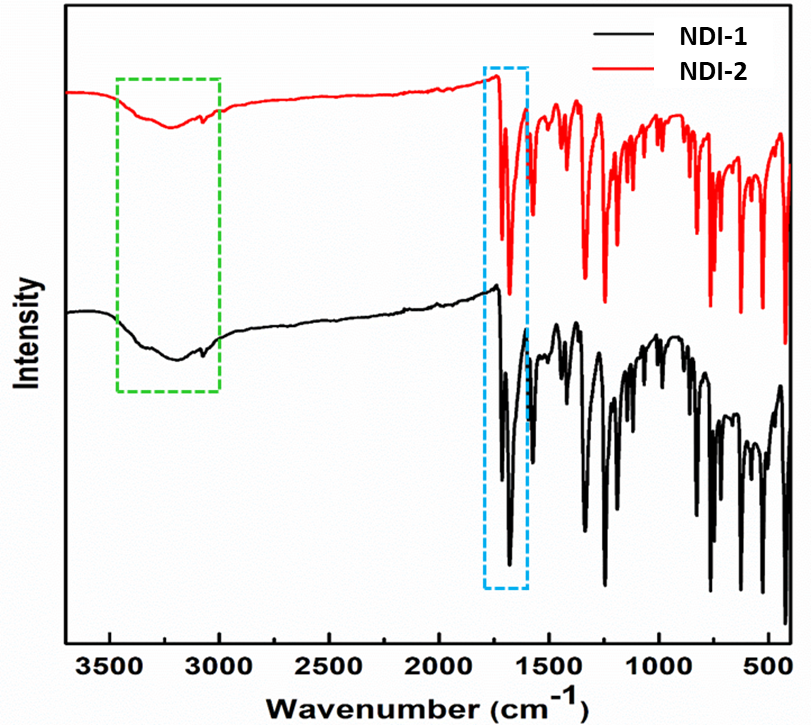


**Figure S1**. FT-IR Spectra of **NDI-1** and **NDI-2**.

**Table S1.** Major FT-IR vibrations of **NDI-1** and **NDI-2.**

| **Bond** | **NDI-1(cm^-1^)** | **NDI-2(cm^-1^)** |
| --- | --- | --- |
| ν(symmetrical, C═O) | 1681 | 1681 |
| ν(asymmetrical, C═O) | 1713 | 1713 |
| ν(–OH) | 3189 | 3218 |
|  |  |  |


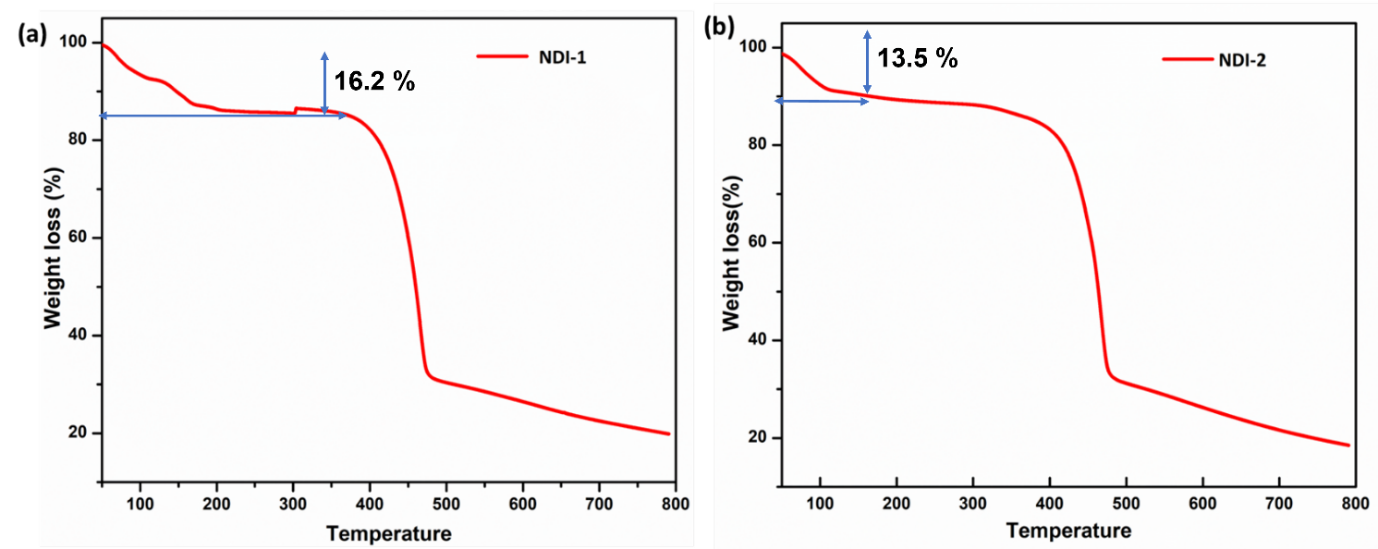


**Figure S2**. Thermogravimetric analysis of **(a)** **NDI-1** and **(b)** **NDI-2**.


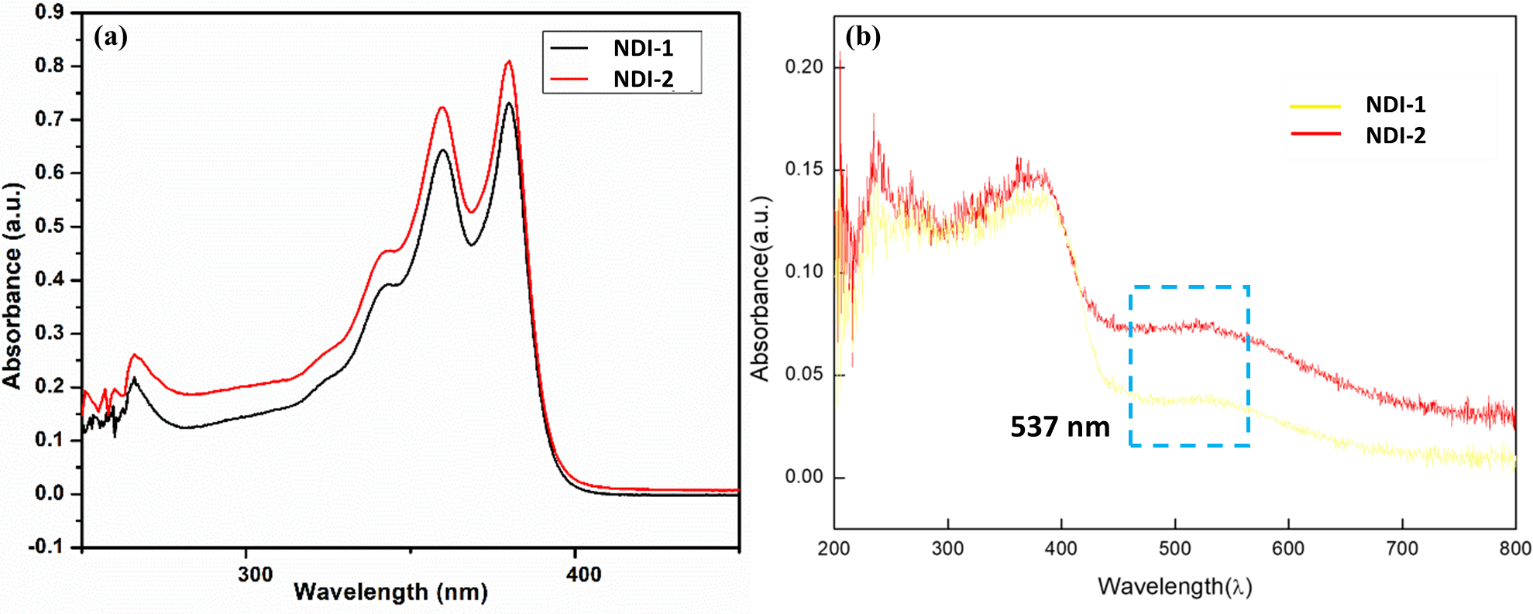


**Figure S3. (a)** UV-visible data in Solution state of **NDI-1** and **NDI-2. (b)** UV-visible data in solid state of **NDI-1** and **NDI-2**.

**
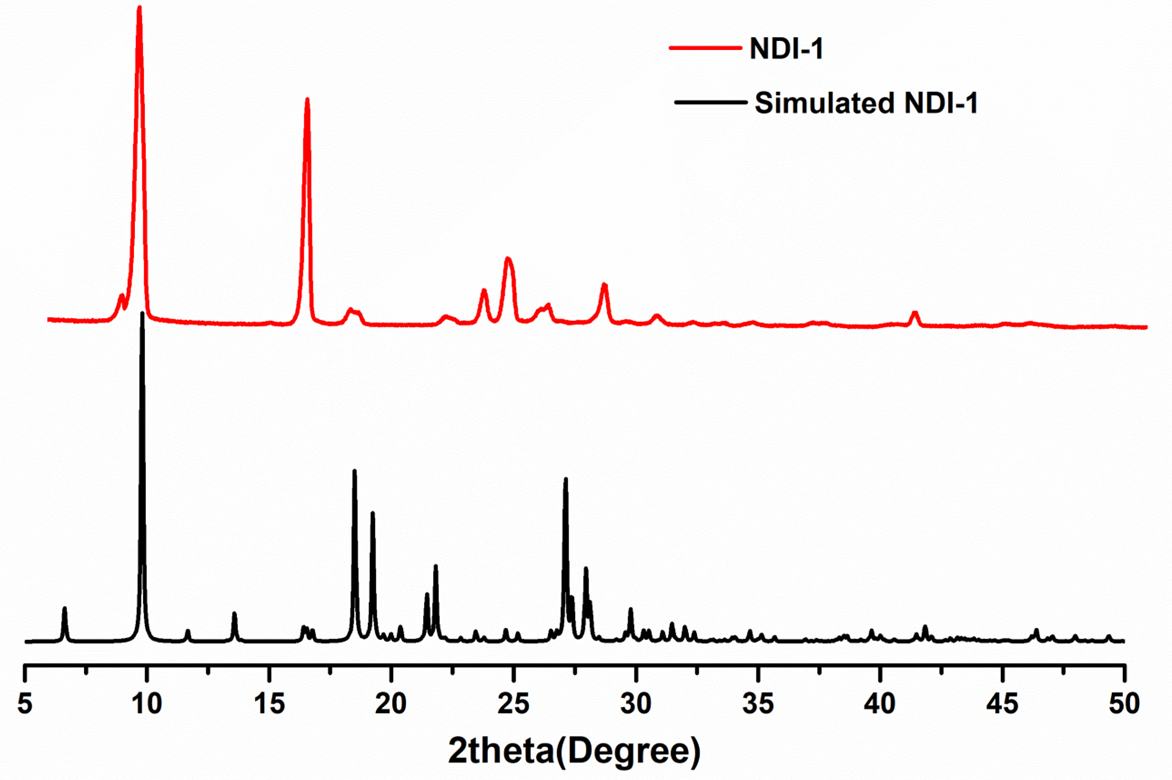
**

**Figure S4.** Powder X-ray Diffraction patterns of Simulated **NDI-1** and synthesized **NDI-1**.

**
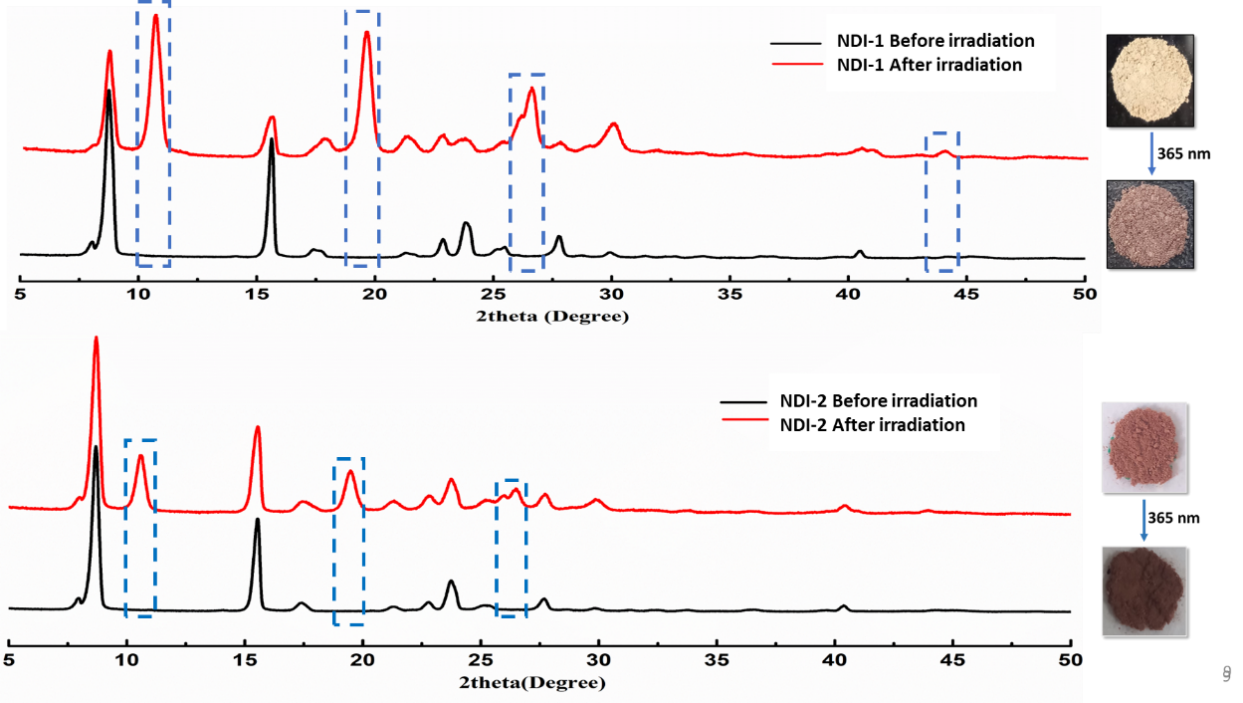
**

**Figure S5.** Powder X-ray Diffraction patterns of **NDI-1** and **NDI-2** before and after irradiation.


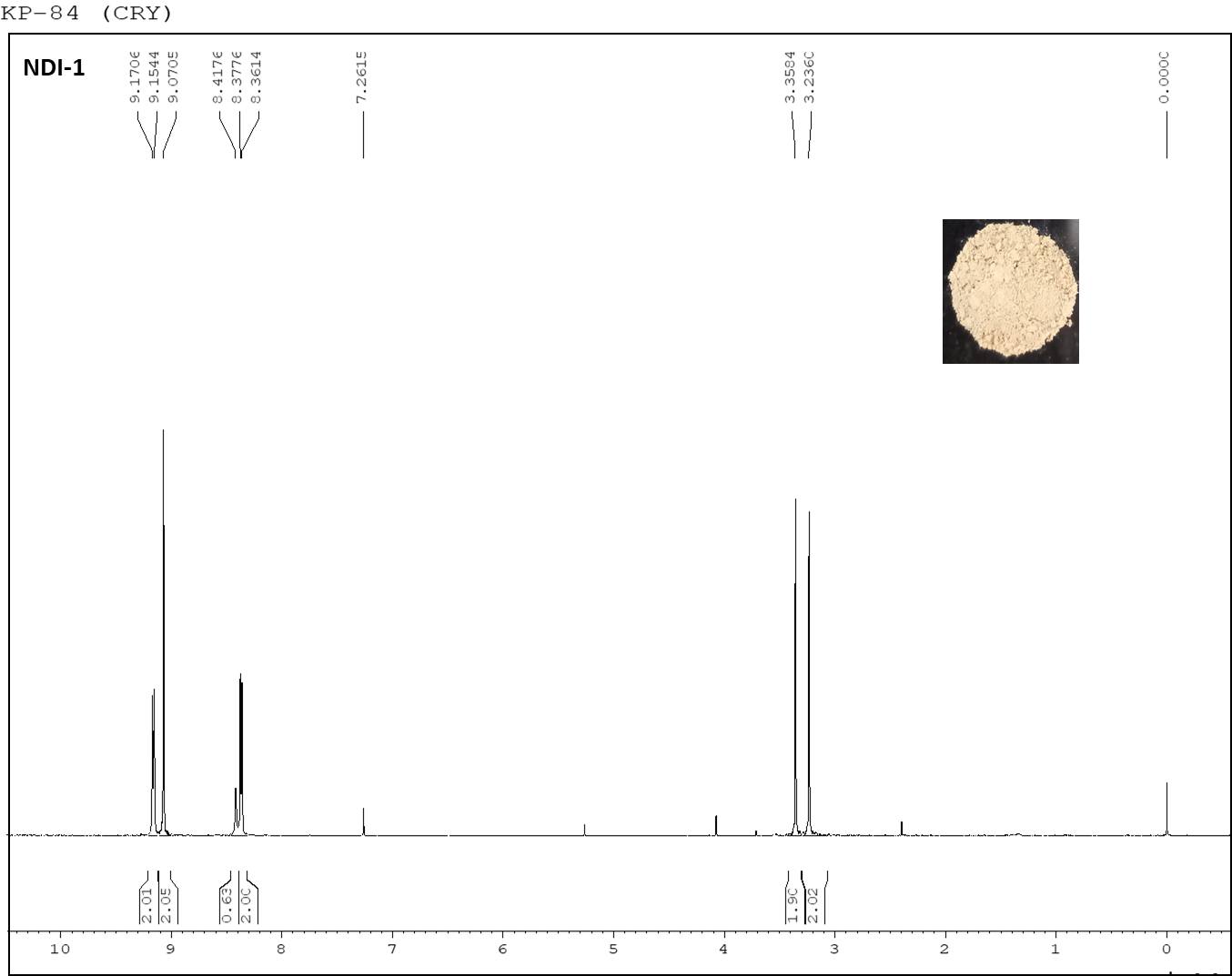


**Figure S6. ^1^**H NMR of **NDI-1**.


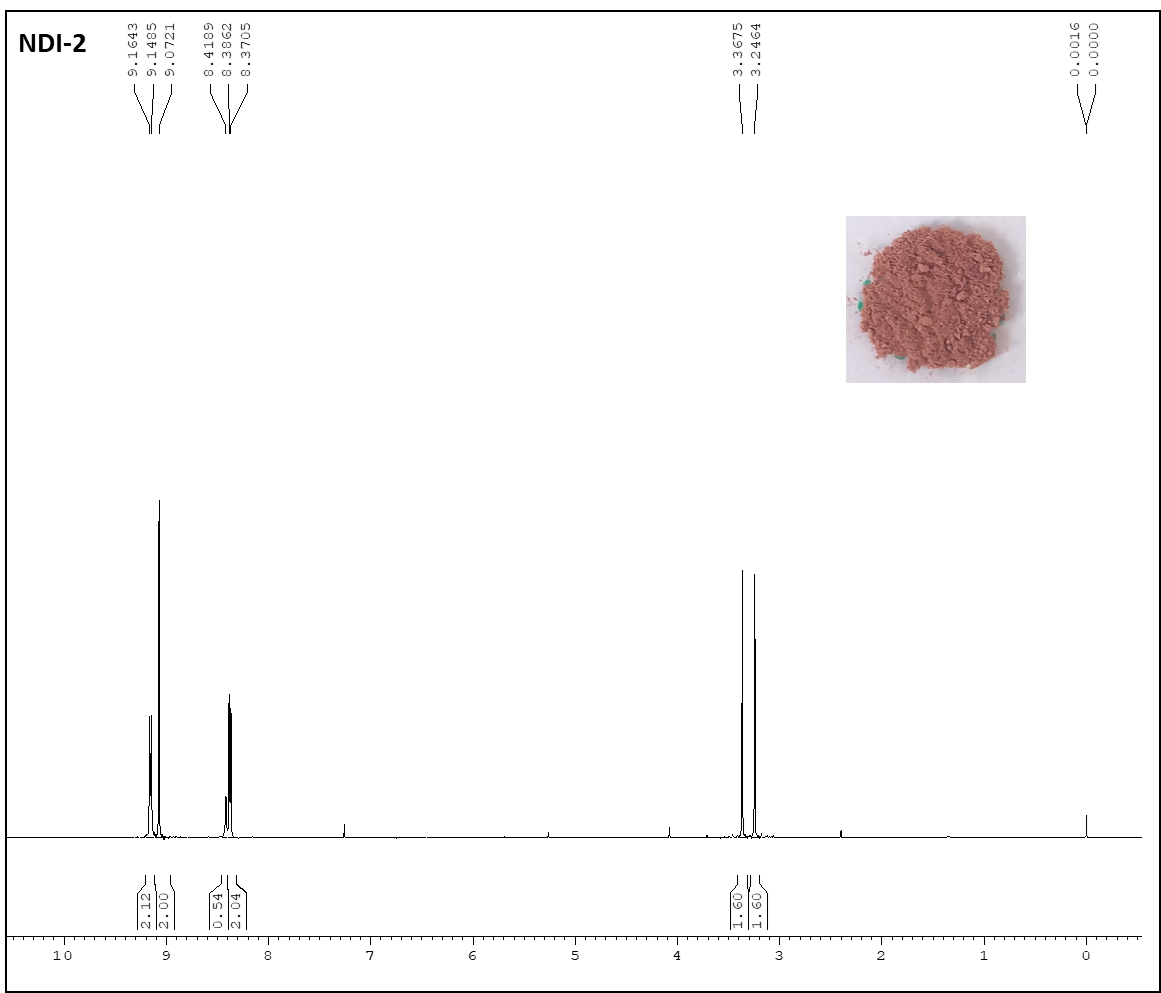


**Figure S7. ^1^**H NMR of **NDI-2**.


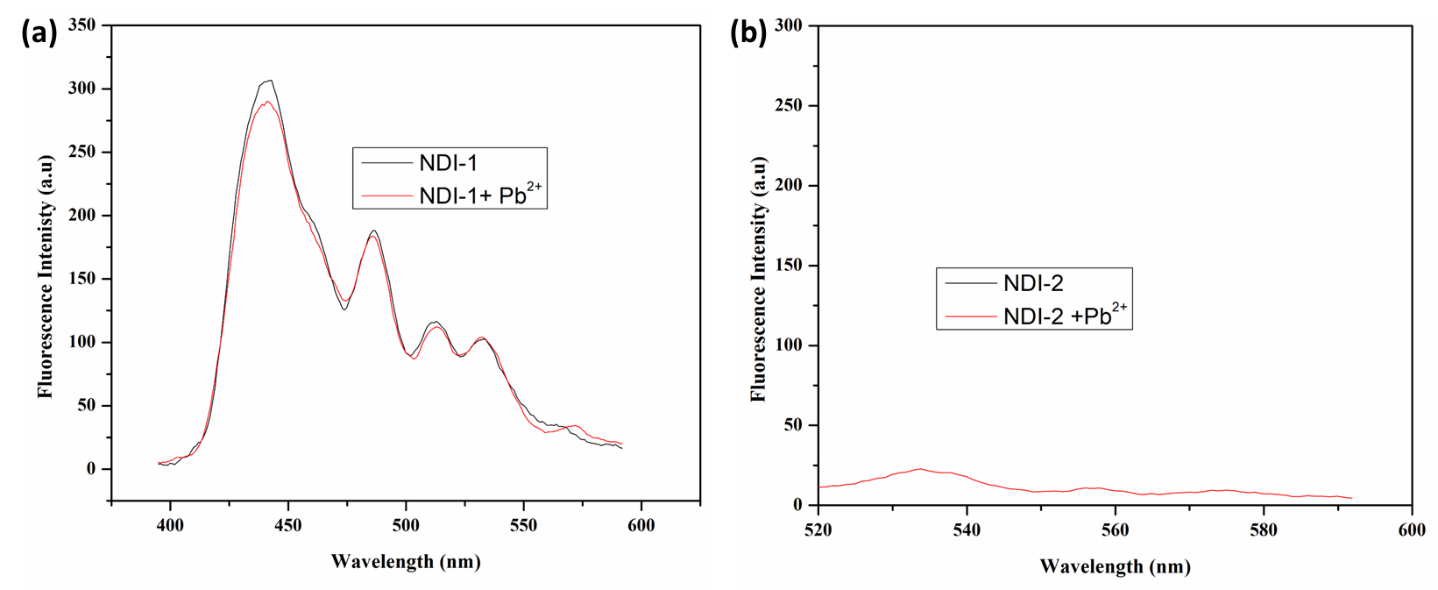


**Figure S8. (a)** Fluorescence spectra of **NDI-1** (10^-5^ M, DMSO) in presence of Pb^2+^ ions (10^-5^ M). **(b)** Fluorescence spectra of **NDI-2** (10^-5^ M, DMSO) in of Pb^2+^ ions (10^-5^ M).


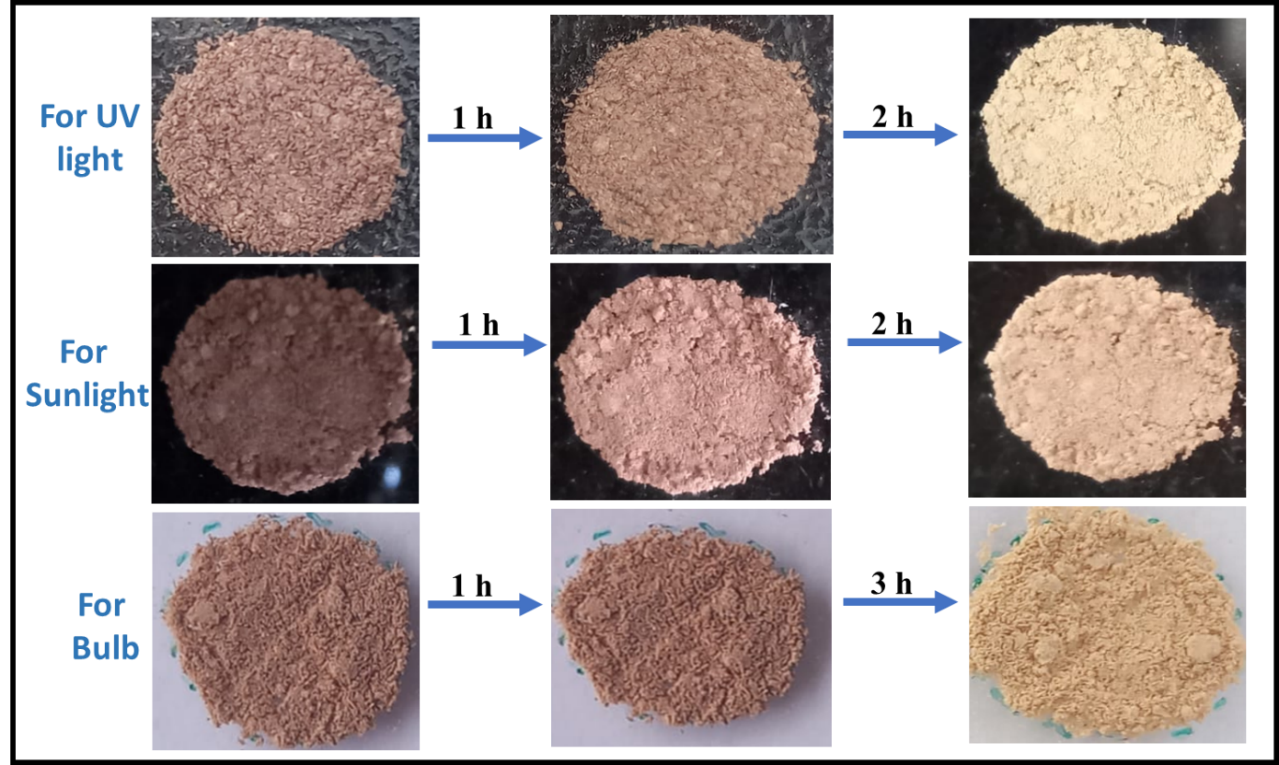


**Figure S9**. Reversible photochromism in **NDI-1** for different light sources.


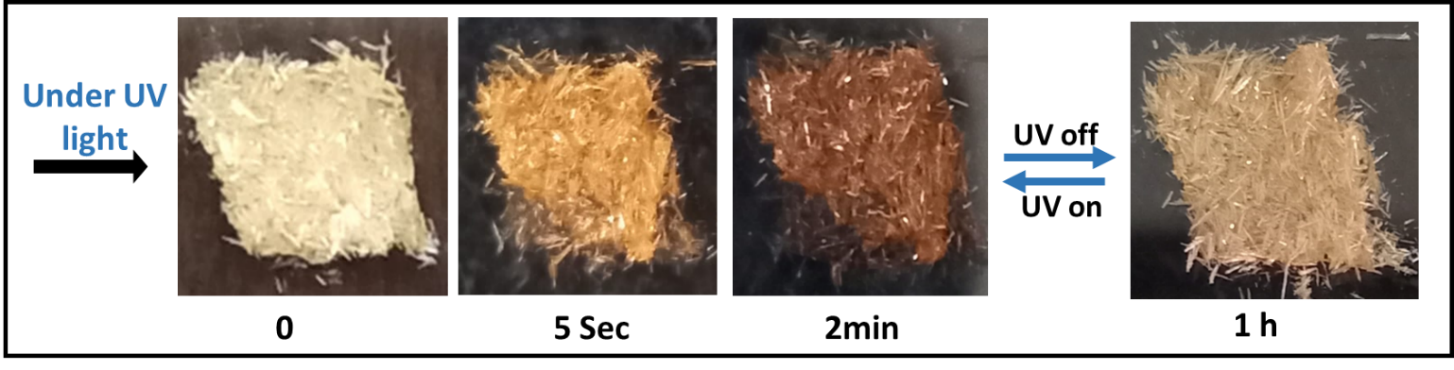


**Figure S10.** Photochromism and its Reversibility in **NDI-1** crystals.


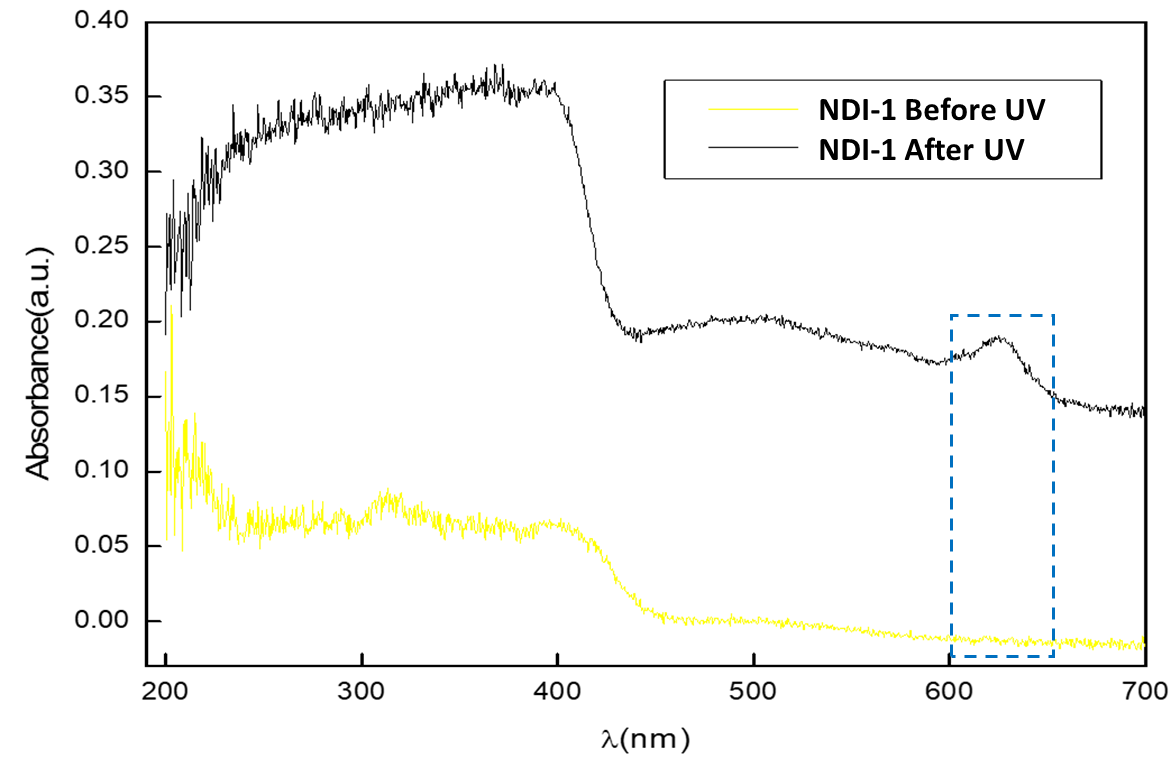


**Figure S11.** Solid state UV absorption data for **NDI-1** crystals before and after irradiation.


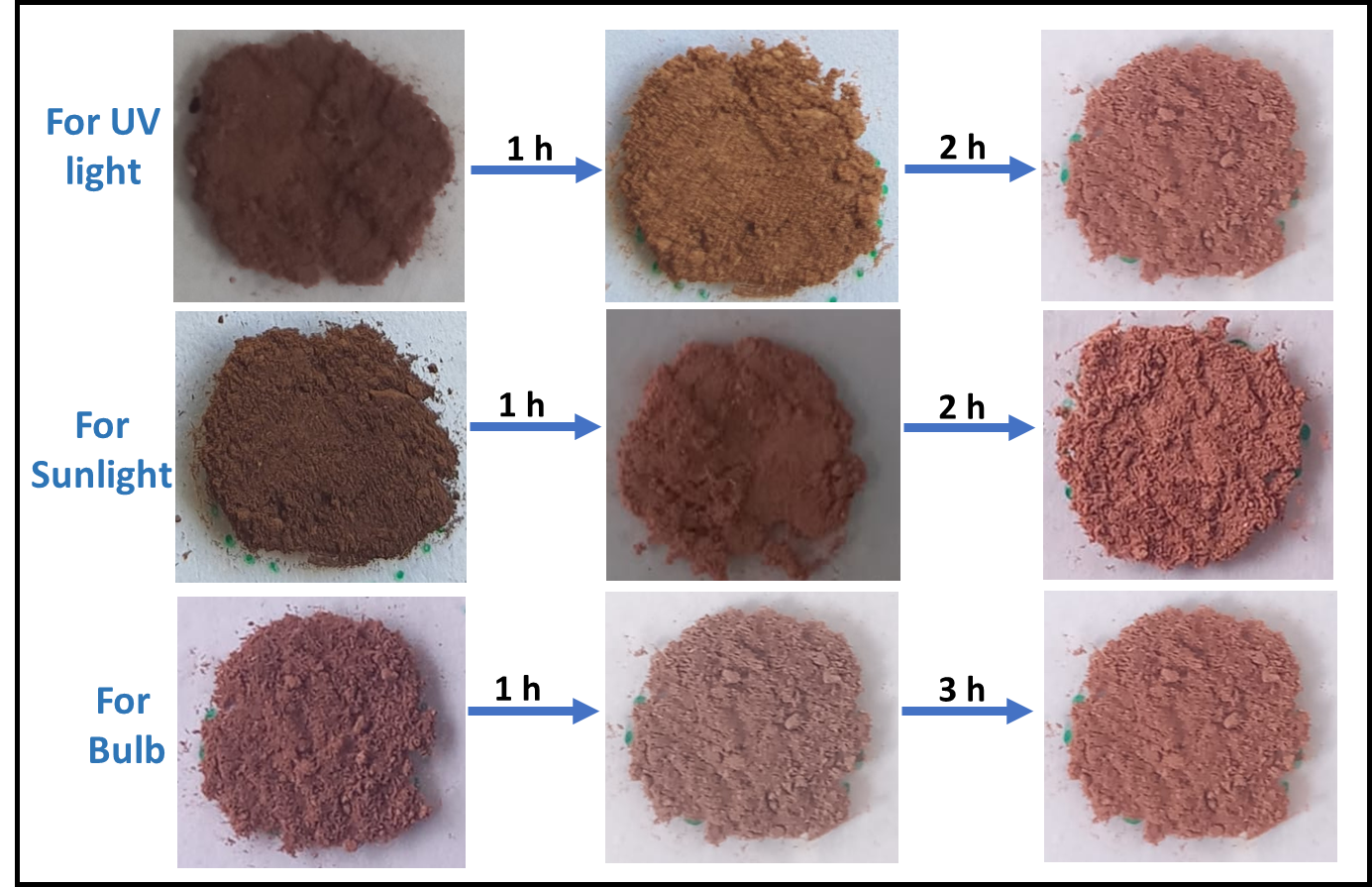


**Figure S12.** Reversible photochromism in **NDI-2** for different light sources.


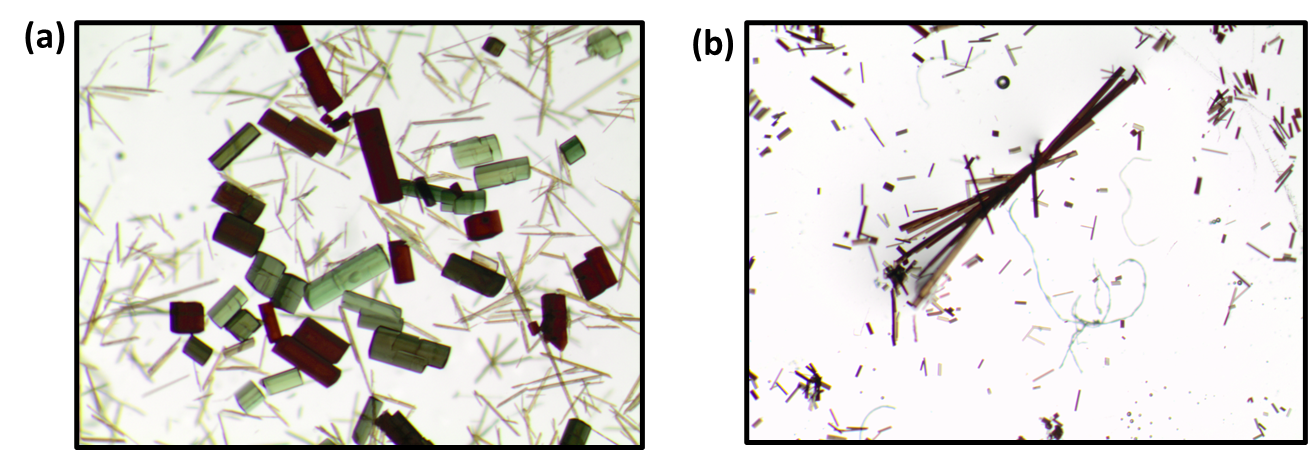


**Figure S13.** Optical images for **(a)** **NDI-1:** block and rod type **(b) NDI-2:** rod type morphology.

**Table S2.** Crystallographic data for synthesized **NDI-1 and NDI-2**

| **Specification** | **NDI-1**  **(CCDC-2512662)** | **NDI-2 (Reported)**  **(CCDC- 1016731)** |
| --- | --- | --- |
| Empirical formula | C27H20N5O6 | C24H12N4O4.2C3H7NO |
| Formula weight | 510.48 | 566.57 |
| Temperature/K | 293(2) | 93.1 |
| Crystal system | Monoclinic | Monoclinic |
| Space group | C2/c | P2_1_/c |
| a, Å | 9.5829(4) | 5.3890(8) |
| b, Å | 26.6297(12) | 12.2012(17) |
| c, Å | 9.2186(4) | 19.998(3) |
| α ° | 90 | 90 |
| β ° | 91.247(4) | 91.764(10) |
| *γ* ° | 90 | 90 |
| Volume Å^3^ | 2351.93(18) | 1314.3(3) |
| Z | 4 | 2 |
| Crystal size/mm^3^ | 0.135**×**0.042×0.037 | 0.50**×**0.10×0.05 |
| ρ (calculated) g/cm^3^ | 1.442 | ------ |
| μ/mm-1 | 0.872 | ------- |
| F(000) | 1060.0 | ------- |
| Radiation | Mo Kα  (λ = 0.71073) | Cu Kα |
| Goodness-of-fit on F^2^ | 1.051 | 0.097 |
| Final R indexes [I>=2σ (I)] | R_1_ = 0.0444, wR_2_ = 0.1172 | R1= 0.097. wR2 = 0.309 |

**Table S3.** Selected bond lengths (Å) for the compound **NDI-1**

| **Atoms** | **Bond Length (Å)** | **Atoms** | **Bond Length (Å)** |
| --- | --- | --- | --- |
| O1 C4 | 1.206 (2) | C5 C6 | 1.371 (2) |
| O2 C11 | 1.212 (2) | C8 C11 | 1.488 (2) |
| N2 C4 | 1.4040 (19) | C8 C7 | 1.374 (2) |
| N2 C3 | 1.448 (3) | C3 C2 | 1.376 (2) |
| N3 C11 | 1.3934 (19) | N1 C1 | 1.332 (3) |
| N3 C12 | 1.451 (3) | C7 C6 | 1.407 (2) |
| N5 C16 | 1.447 (3) | N4 C14 | 1.326 (3) |
| N5 C15 | 1.335 (4) | C12 C13 | 1.373 (2) |
| C9 C10 | 1.415 (3) | C2 C1 | 1.382 (3) |
| C9C8 | 1.410 (2) | C15 O4 | 1.186 (4) |
| C10 C5 | 1.4138 (19) | C13 14 | 1.386 (3) |
| C5 C4 | 1.488 (2) |  |  |

**Table S4.** Selected bond angles (Å) for the compound **NDI-1**

| **Atoms** | **Bond Angles (Å)** | **Atoms** | **Bond Angles (Å)** |
| --- | --- | --- | --- |
| C4 N2 C3 | 126.07 (18) | O2 C11 C8 | 122.52 (16) |
| C11 N3 C12 | 116.97 (9) | N3 C11 C8 | 116.45 (14) |
| C15 N5 C16 | 121.26 (13) | O1 C4 N2 | 120.91 (15) |
| C8 C9 C10 | 119.06 (10) | O1 C4 C5 | 122.97 (15) |
| C5 C10 C9 | 119.11 (10) | N2 C4 C5 | 116.11 (14) |
| C10 C5 C4 | 119.46 (15) | C2 C3 N2 | 119.74 (12) |
| C6 C5 C10 | 120.57 (16) | C8 C7 C6 | 120.07 (15) |
| C6 C5 C4 | 119.91 (15) | C5 C6 C7 | 120.35 (15) |
| C9 C8 C11 | 119.35 (15) | C13 C12 N3 | 119.82 (12) |
| C7 C8 C9 | 120.75 (16) | C3 C2 C1 | 117.3 (2) |
| C7 C8 C11 | 119.90 (15) | O4 C15 N5 | 126.0 (2) |
| O2 C11 N3 | 121.03 (16) | C12 C13 C14 | 117.4 (2) |
| N1 C1 C2 | 123.9 (2) | N4 C14 C13 | 123.8 (2) |


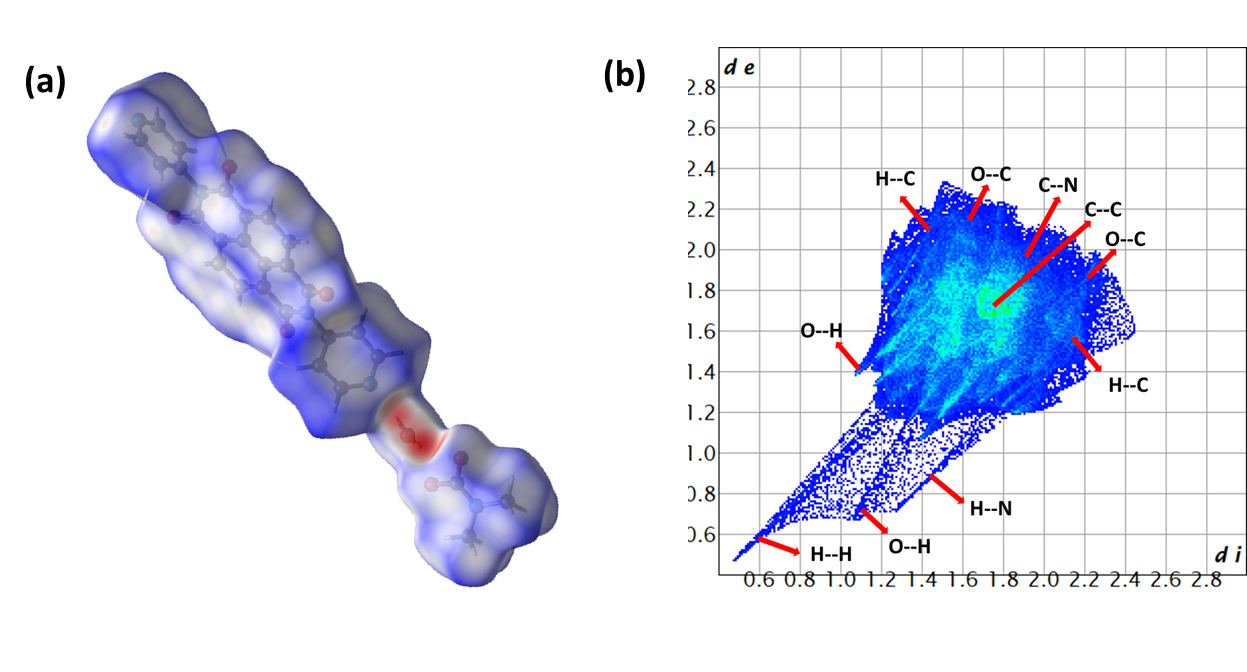


**Figure S14.** **(a)** Hirshfeld surface analysis of **NDI-1**. **(b)** Hirshfeld 2D fingerprint plot of **NDI-1**.


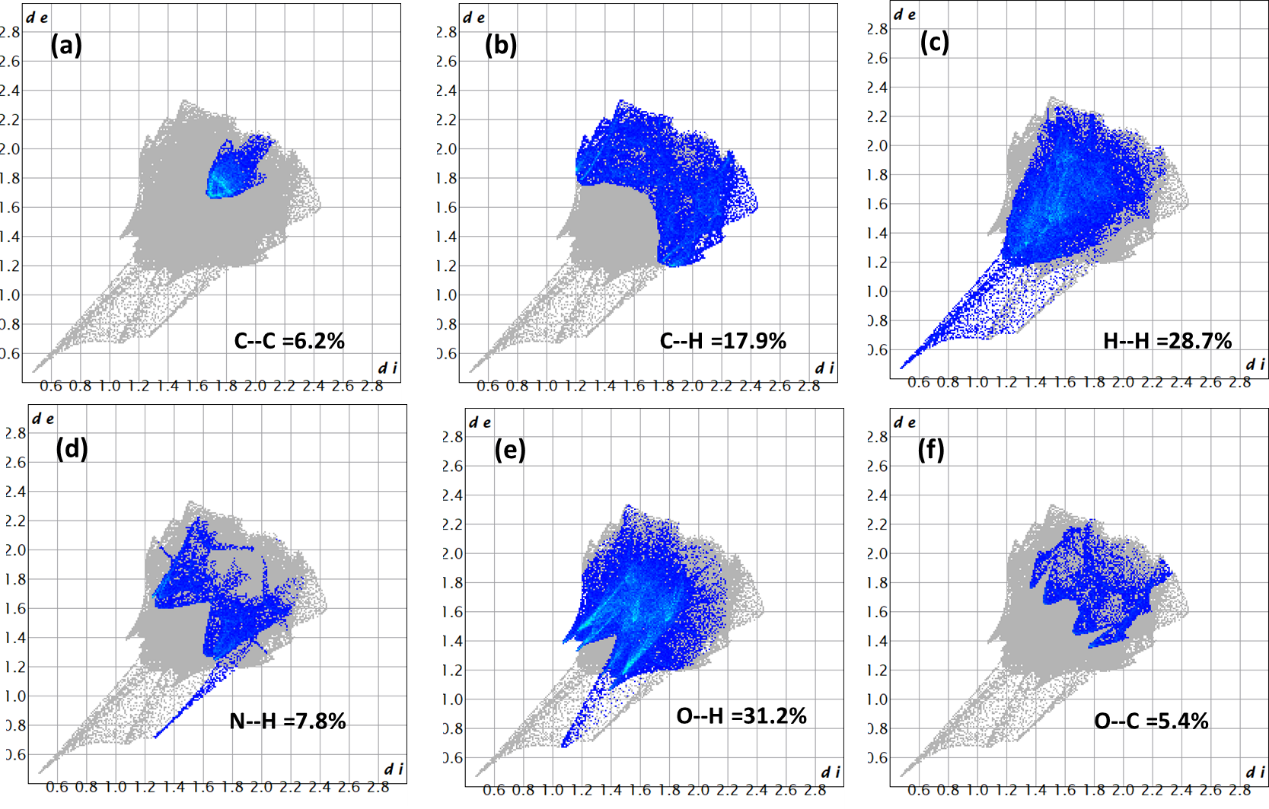


**Figure S15.** **(a-f)** 2D fingerprint plots of **NDI-1** to obtain quantitative non-covalent interactions
outlined from Hirshfeld surface analysis. The grey part in 2D finger plots indicates the total interactions calculated from *d_norm_*.


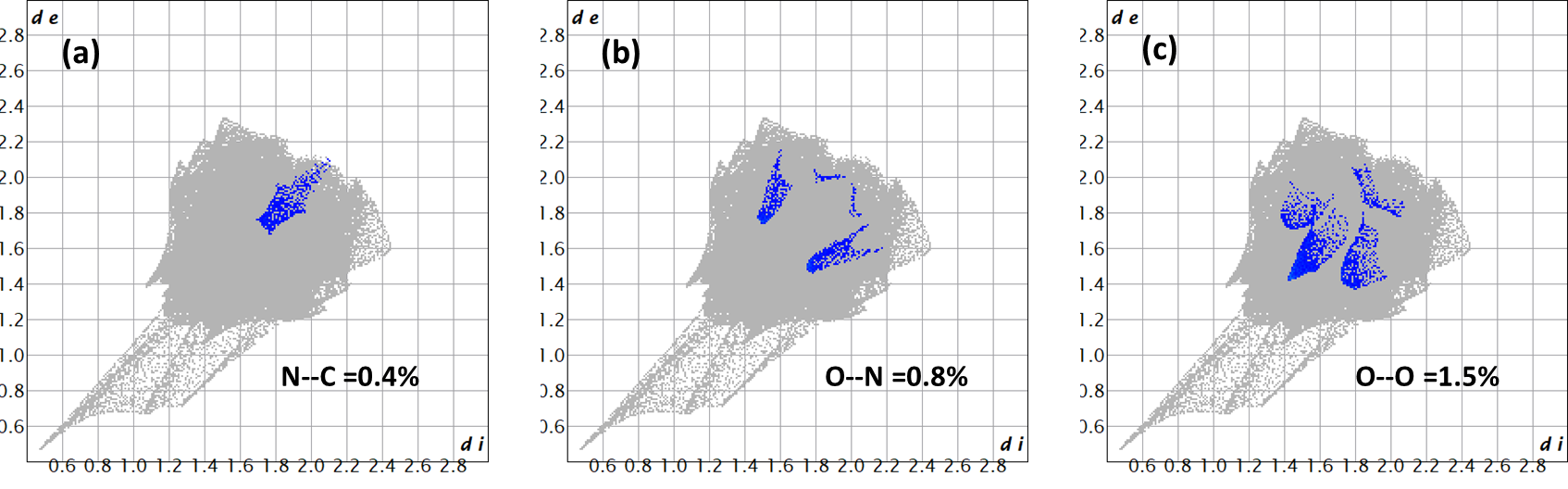


**Figure S16**. **(a-c)** 2D fingerprint plots of N–C**,** O–N**,** and O–O for **NDI-1** to obtain quantitative non-covalent interactions. The grey part in 2D finger plots indicates the total interactions calculated from *d_norm_*.


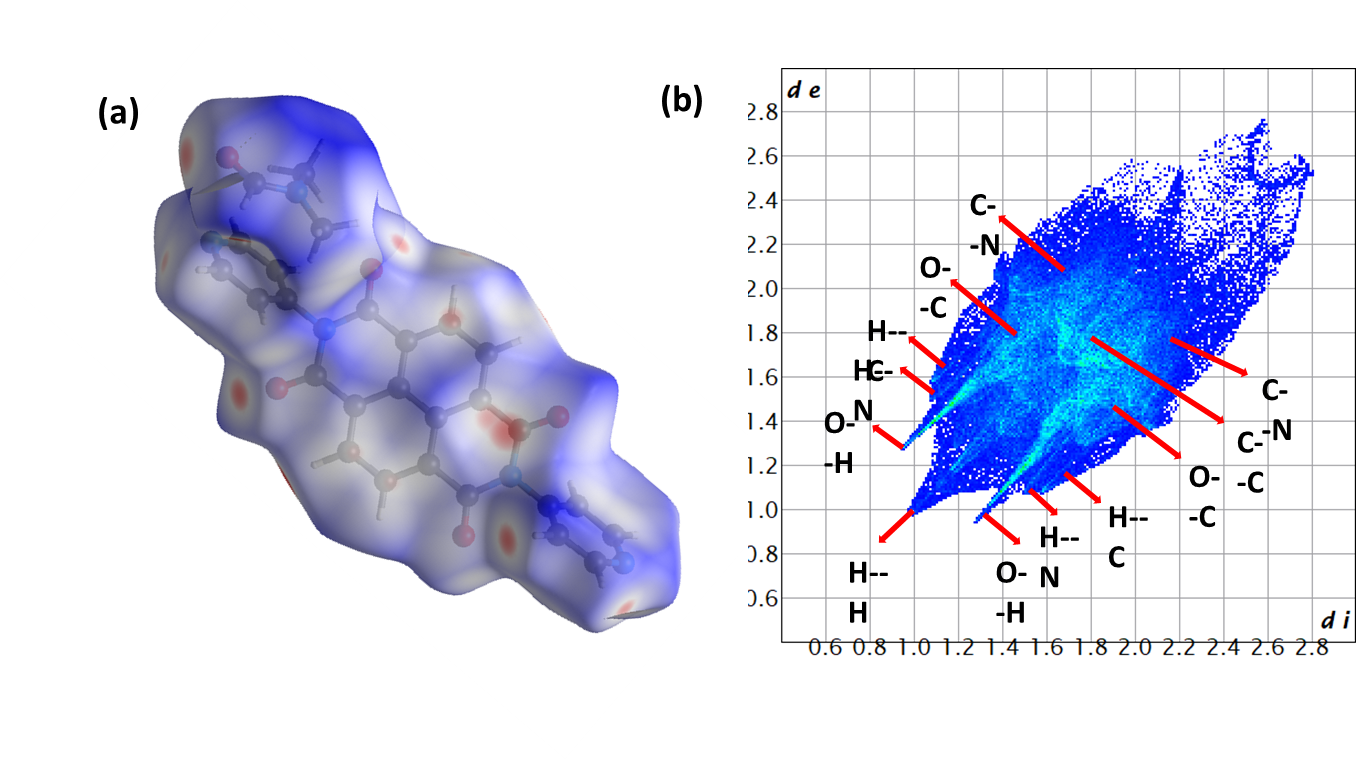


**Figure S17.** **(a)** Hirshfeld surface analysis of **NDI-2**. **(b)** Hirshfeld 2D fingerprint plot of **NDI-2**.


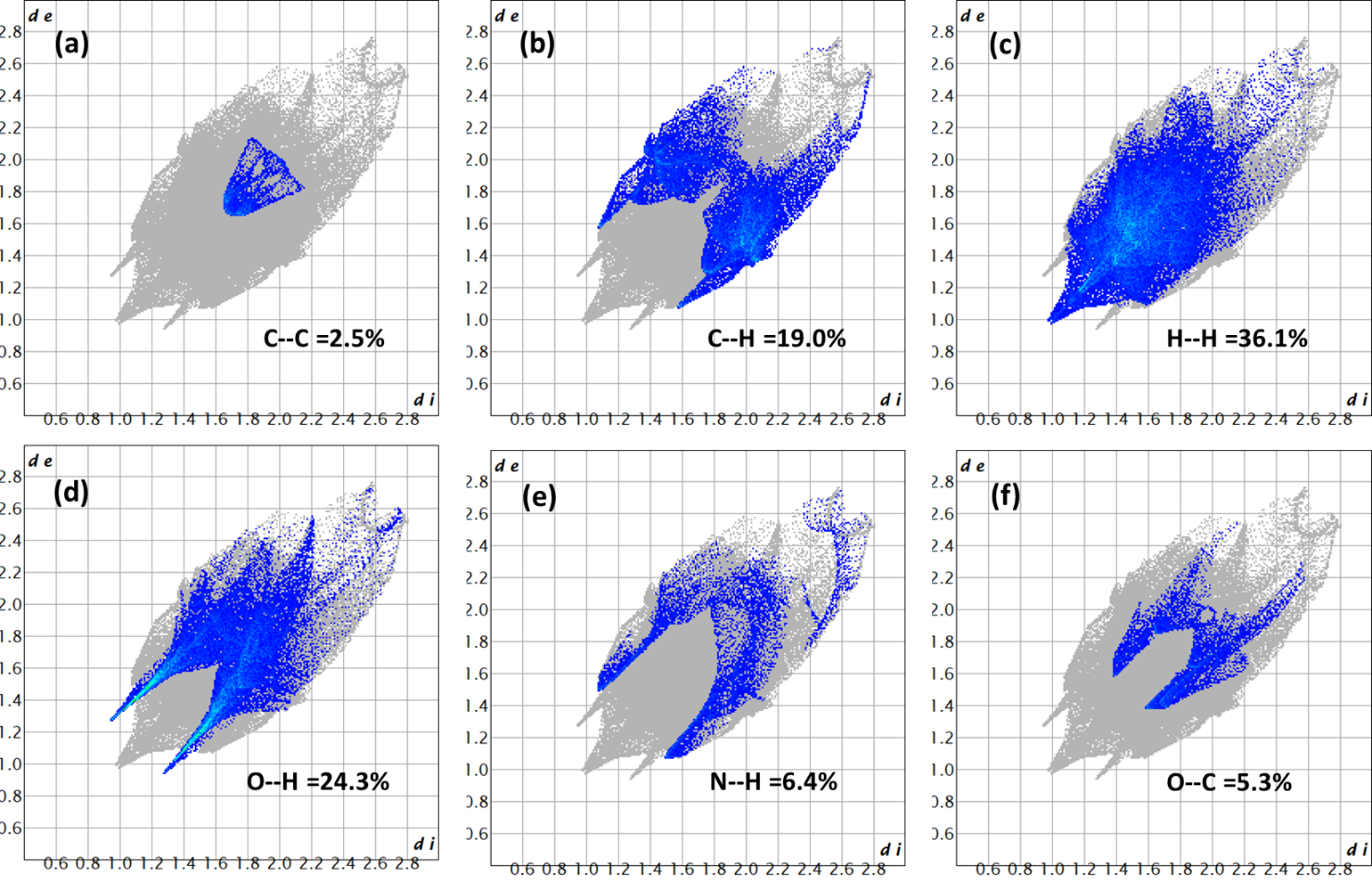


**Figure S18.** **(a-f)** 2D fingerprint plots of **NDI-2** to obtain quantitative non-covalent interactions
outlined from Hirshfeld surface analysis. The grey part in 2D finger plots indicates the total interactions calculated from *d_norm_*.


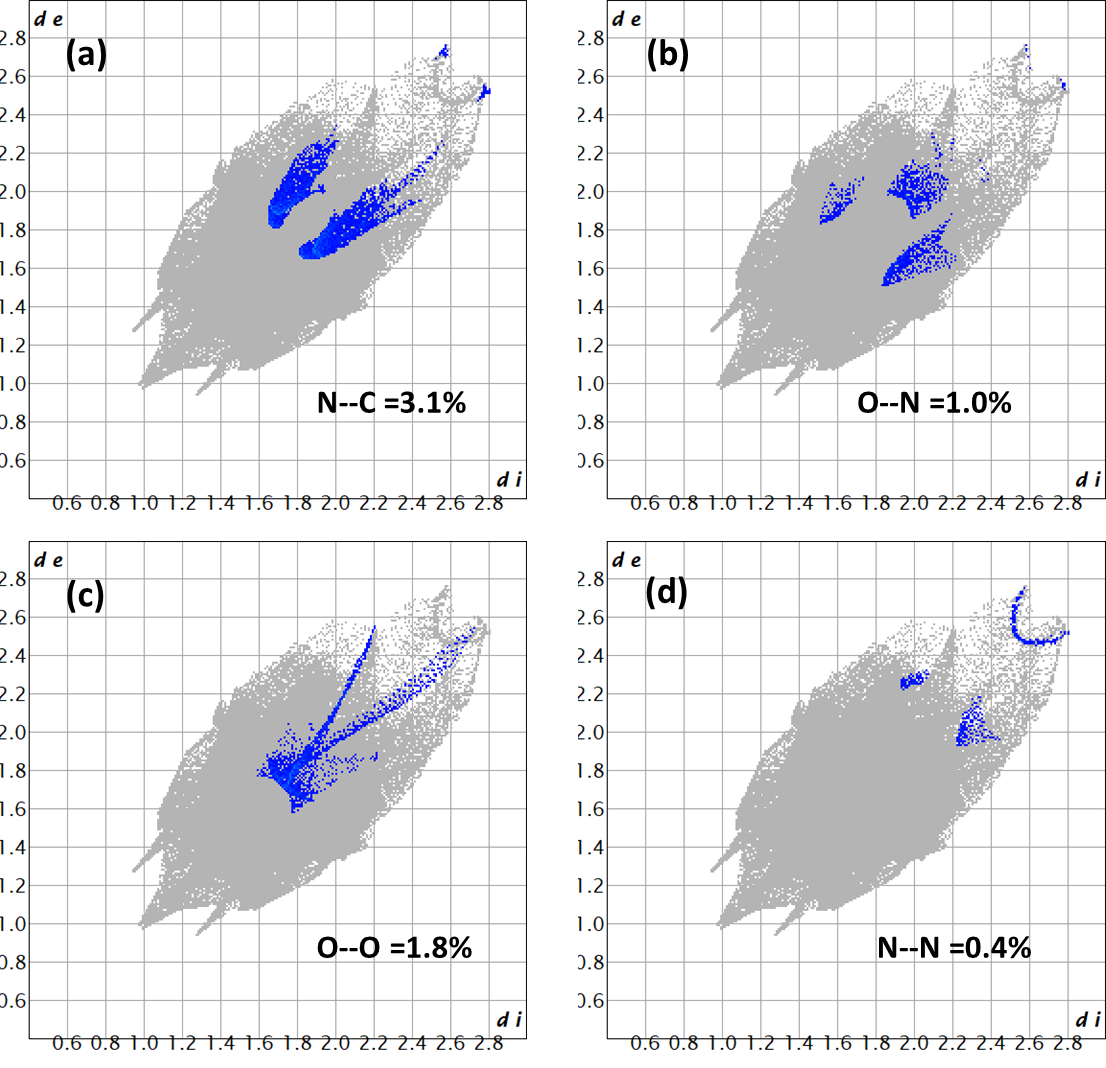


**Figure S19**. **(a-c)** 2D fingerprint plots of N–C**,** O–N**,** O–O, and N–N for **NDI-2** to obtain quantitative non-covalent interactions. The grey part in 2D finger plots indicates the total interactions calculated from *d_norm_*


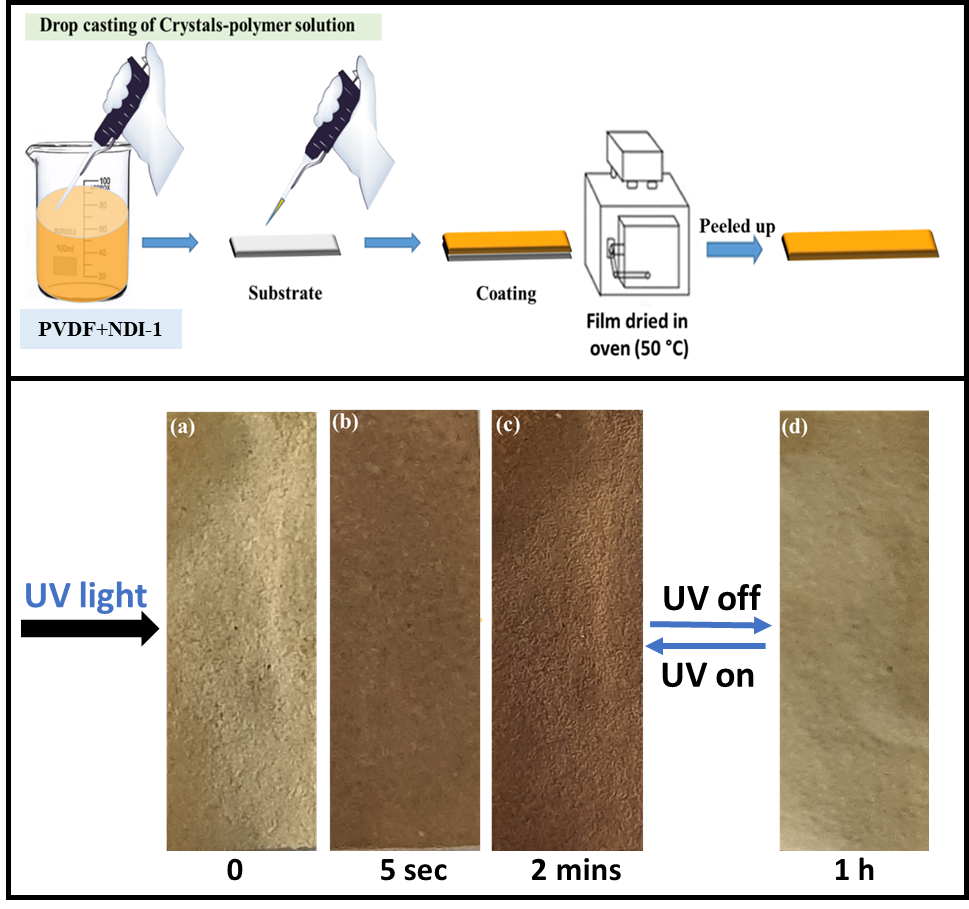


**Scheme S1.** Synthesis of Crystal polymer composite of **NDI-1** using PVDF (Polyvinyldene fluoride) Polymer and its Photochromic behaviour.


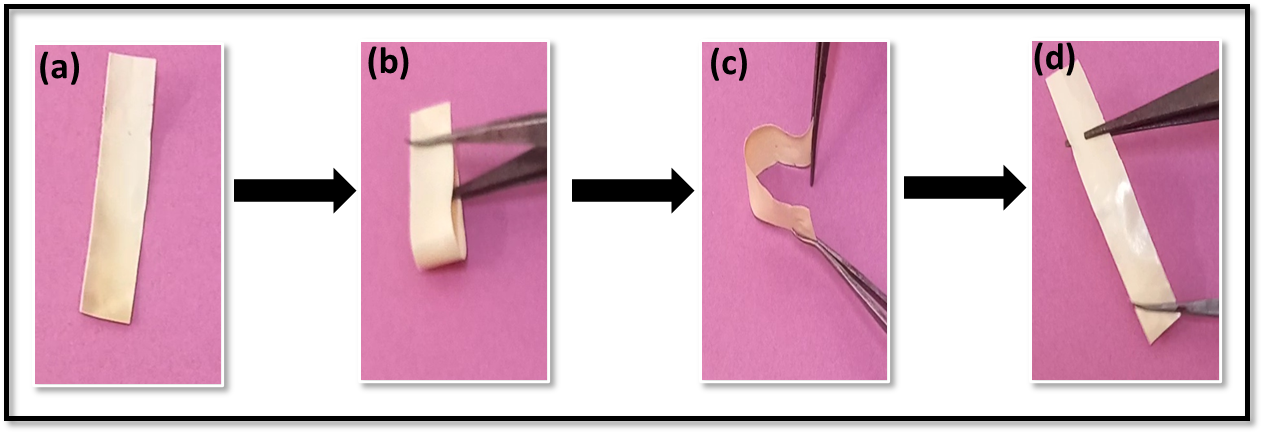


**Figure S20.** The composite film **PVDF@NDI-1** showing mechanical flexibility under mechanical stress.
